# Hormetic and synergistic effects of cancer treatments revealed by modelling combinations of radio - or chemotherapy with immunotherapy

**DOI:** 10.1186/s12885-023-11542-6

**Published:** 2023-10-27

**Authors:** Sanyi Tang, Shuo Li, Biao Tang, Xia Wang, Yanni Xiao, Robert A. Cheke

**Affiliations:** 1 School of Mathematics and Statistics, Shaanxi Normal University, Xi’an, 710119 People’s Republic of China; 2 The Interdisplinary Research Center for Mathematics and Life Sciences, Xi’an Jiaotong University, Xi’an, 710049 People’s Republic of China; 3 Natural Resources Institute, University of Greenwich at Medway, Central Avenue, Chatham Maritime, Kent, ME4 4TB UK; 4 Department of Infectious Disease Epidemiology, School of Public Health, Imperial College London, St Mary’s Campus, Norfolk Place, London, W2 1PG UK

**Keywords:** Tumour, Immune response curve, Radio-chemotherapy response curve, Mixed response curve, Hormesis, Synergy

## Abstract

**Background:**

Radio/chemotherapy and immune systems provide examples of hormesis, as tumours can be stimulated (or reduced) at low radio/chemical or antibody doses but inhibited (or stimulated) by high doses.

**Methods:**

Interactions between effector cells, tumour cells and cytokines with pulsed radio/chemo-immunotherapy were modelled using a pulse differential system.

**Results:**

Our results show that radio/chemotherapy (dose) response curves (RCRC) and/or immune response curves (IRC) or a combination of both, undergo homeostatic changes or catastrophic shifts revealing hormesis in many parameter regions. Some mixed response curves had multiple humps, posing challenges for interpretation of clinical trials and experimental design, due to a fuzzy region between an hormetic zone and the toxic threshold. Mixed response curves from two parameter bifurcation analyses demonstrated that low-dose radio/chemotherapy and strong immunotherapy counteract side-effects of radio/chemotherapy on effector cells and cytokines and stimulate effects of immunotherapy on tumour growth. The implications for clinical applications were confirmed by good fits to our model of RCRC and IRC data.

**Conclusions:**

The combination of low-dose radio/chemotherapy and high-dose immunotherapy is very effective for many solid tumours. The net benefit and synergistic effect of combined therapy is conducive to the treatment and inhibition of tumour cells.

**Supplementary Information:**

The online version contains supplementary material available at 10.1186/s12885-023-11542-6.

## Introduction

**Fig. 1:**
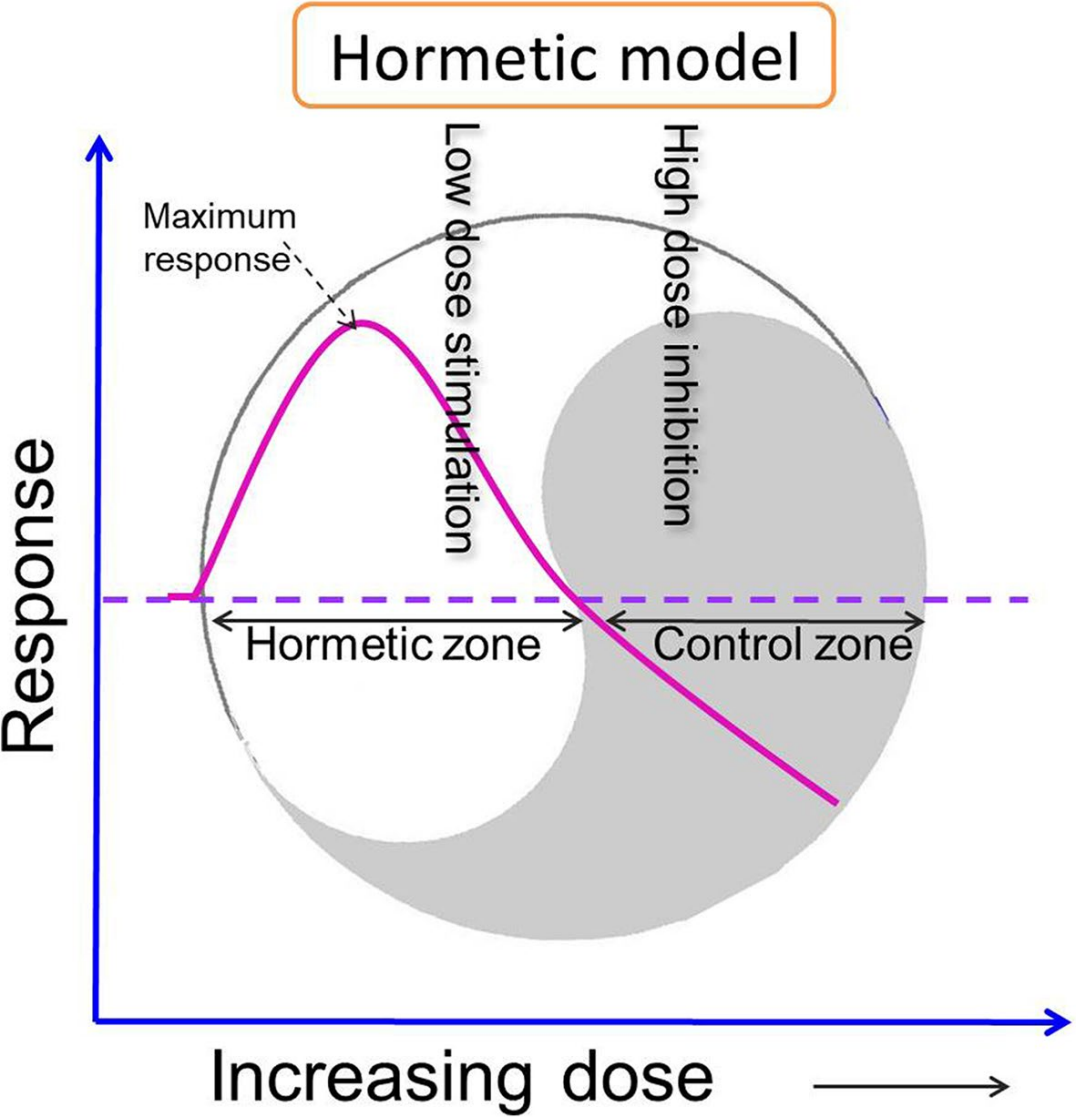
Quantitative features of a typical dose–response curve displaying hormesis, illustrating the maximum stimulatory response, hormetic and control zones and the toxic threshold

As the growth and inhibition mechanisms of cancer cells are still not fully understood, cancer remains one of the leading causes of death in the world. Therefore, a variety of therapeutic measures against tumours have been proposed, the most typical of which are radiotherapy, chemotherapy, immunotherapy, virus therapy or combinations of two or more of these treatments [1–3]. Immunotherapy and radio/chemotherapy have different ways of targeting tumour cells, but their combined effects are proven to be more effective [1, 4–6]. Mathematical models of interactions between tumour cells, immune cells and cytokines, as described in this paper, are valuable tools for analysing and verifying the effectiveness of single treatment or combined treatment strategies [5, 7–9]. Note that the treatments can additionally interfere with the proliferation of normal cells, amongst which are immune components [10, 11]. Thus, strong radio- and/or chemotherapy is sometimes assumed to be ineffective when combined with immunotherapy because of the possible negative effects of such treatments on immune systems [10, 12]. Furthermore, inappropriate radio/chemotherapy, immunotherapy or even combined therapy can produce paradoxical and hormetic effects [13–20]. Hormesis is the phenomenon in which small doses of an intervention such as radiation, drugs or immune reactants show stimulatory effects on targets, while high doses show inhibitory effects, thus posing significant challenges for decision-making in therapies of cancer [17–25]. The quantitative features of an hormetic model are shown in Fig. 1.

Radiation hormesis and the implications of low-dose radiation applications in cancer risk assessment have been investigated by numerous researchers [10–12, 20]. Chemotherapy, involving drugs to kill cancer cells, often induces a rapid reduction in tumour size, followed by re-growth. Therefore, metronomic chemotherapy with low-dose, well-tolerated, intermittent chemotherapy has been developed and proposed to replace the conventional high-dose approach [6, 19]. However, chemotherapy can also have hormetic effects [17]. For convenience, such dualistic roles of radio/chemotherapy on tumour cells, resulting in bi-phasic dose responses, are here defined as the radio/chemotherapy response curve (RCRC). The immune response to cancer is a double-edged sword since on the one hand it is involved in immunosurveillance of the tumour but on the other hand it can stimulate tumour-promoting inflammation. This means that there are some tumour directed immune reactants, anti-cancer antibodies, which stimulate tumour growth at low doses but inhibit such growth at higher doses. Thus, anti-cancer agents possess a bi-phasic sigmoidal dose response relationship consisting of hormetic and cytotoxic effects [17–19, 21–24], referred to here as the immune response curve (IRC).

An effective way to solve these challenging problems is to use combination therapy: combining radio/chemotherapy with immunotherapy can be a promising and synergistic option to treat many cancer types [1, 4, 5], including lung cancer or gastric cancer treatment. However, such a strategy will produce what we are calling here a mixed response curve (MRC), i.e. a mixed bi-phasic sigmoidal dose response [1, 5, 26]. Before the clinical roles of RCRCs and IRCs in tumour treatment are fully understood, we need to know what the MRC will mean for clinical trials and what challenges and net effects it will bring to tumour therapy. In order to discuss these issues in depth, we employ the classic kinetic model of interactions between tumour cells, immune cells and cytokines [7, 8], and introduce pulsed radio-/chemotherapy together with immunotherapy [8, 27, 28]. The threshold conditions for the existence and stability of a tumour free periodic solution are provided, and this provides a basis for decisions on the design of treatment strategies aimed at eradicating tumour cells.

We used values for the parameters from the literature and numerical bifurcation analysis techniques to reveal the parameter space and the mechanism of the paradoxical and hormetic effects produced by radio- and/or chemotherapy, immunotherapy and combination therapy. The main results revealed that the RCRC, IRC and MRC undergo homeostatic changes or catastrophic shifts and show hormetic effects in many parameter regions. The resultant discoveries of a “mixed fuzzy region” between the hormetic zone and the toxic threshold and MRCs with multiple humps bring new challenges for the design and interpretation of clinical trials of anti-tumour therapies. For combination therapy, the main conclusion is that low-dose radio/chemotherapy and high-dose immunotherapy can effectively inhibit the growth of tumour cells, or even eradicate them. The results have implications for potential clinical applications, which we have confirmed by fitting our model to RCRC and IRC data sets.

## Methods

### Model

We modelled the interactions between effector cells ($E$), tumour cells ($T$) and a cytokine (interleukin 2, IL-2,${I}_{L}$) with pulsed radio/chemotherapy and immunotherapy with the following equations [7, 8, 27, 28]:

1 $$\left\{\begin{array}{c}\frac{dE\left(t\right)}{dt}=cT-{\mu }_{2}E+\frac{{p}_{1}E{I}_{L}}{{g}_{1}+{I}_{L}}, \\ \frac{dT\left(t\right)}{dt}={r}_{2}\left(T\right)T-\frac{aET}{{g}_{2}+T}, t\ne nP, \\ \frac{d{I}_{L}\left(t\right)}{dt}=\frac{{p}_{2}ET}{{g}_{3}+T}-{\mu }_{3}{I}_{L}, \\ E\left({t}^{+}\right)=\left(1-{q}_{1}\right)E\left(t\right)+{s}_{1}, \\ T\left({t}^{+}\right)=\left(1-{q}_{2}\right)T\left(t\right), t=nP. \\ {I}_{L}\left({t}^{+}\right)=\left(1-{q}_{3}\right){I}_{L}\left(t\right)+{s}_{2}\end{array}\right.$$

Model 1 consists of two parts: the first part, three differential equations, describes the dynamic evolution of the interaction of $E,$ $T$ and ${I}_{L}$ with the logistic growth function ${r}_{2}\left(T\right)={r}_{2}(1-bT)$. The second part, consisting of three impulsive maps, describes the effects of pulses of radio/chemo-immunotherapy applied at period $P(n=\mathrm{1,2},\cdots$) on the $E,$ $T$ and ${I}_{L}$. ${s}_{1}$ is a treatment term that represents an external source of effector cells such as LAK or TIL cells, and ${s}_{2}$ is a treatment term that represents an external input of cytokines into the system. See reference (7) and Sect. 1 of Supplementary Material for explanations of all the parameters in more detail.

### Threshold conditions for the tumour-free periodic solution

The ideal outcome of integrated therapies against tumours is to eradicate the tumour cells. This can be realized, from a mathematical point of view, provided that model (1) exists with a stable periodic solution, denoted by $\left({E}^{P}\left(t\right),0, {I}_{L}^{P}(t)\right)$, where *E* denotes the number of effector cells and *I*_*L*_ the quantity of cytokines (interleukin 2), both at period *P*. To determine if this is possible, let the number of tumour cells $T\left(t\right)=0$ and consider the subsystem (S2.2) for $E\left(t\right) \mathrm{and} {I}_{L}(t)$, and it follows from Sect. 2 of the Supplementary Material that if

$${\uplambda }_{1}=\left(1-{q}_{1}\right)\mathrm{exp}\left[-{\mu }_{2}P-\frac{{p}_{1}}{{\mu }_{3}}\mathrm{ln}\left(\frac{{g}_{1}+{I}_{L}^{*}{ e}^{-{\mu }_{3}P}}{{g}_{1}+{I}_{L}^{*}}\right)\right]<1$$

then the subsystem exists with a globally stable periodic solution $\left({E}^{P}\left(t\right), {I}_{L}^{P}(t)\right)$, as shown in Fig.S.1a-b and Fig.S.2c-d. Theoretically, we show that if the threshold condition ${\lambda }_{1}>1$ then the impulsive point series $E\left(n{P}^{+}\right)\triangleq {E}_{n}^{P+}$ will tend to infinity, and consequently the solution $E(\mathrm{t})$ will tend to infinity(Fig.S.1c and d.). However, ${I}_{L}(t)$ will finally tend to the periodic solution ${I}_{L}^{P}(t)$ which exists naturally if immunotherapy is applied, i.e.${s}_{2}>0$, as shown in Fig. S.2a and b. This property reveals that we should design an immunotherapy strategy to avoid redundancy, i.e. by keeping ${\lambda }_{1}<1$ during the therapy. Further if

$${\uplambda }_{2}=\left(1-{q}_{2}\right)\mathrm{exp}\left[{r}_{2}(0)P-{\int }_{0}^{P}\frac{a{E}^{P}\left(t\right)}{{g}_{2}}dt\right]<1$$

then the tumour-free periodic solution $\left({E}^{P}\left(t\right),0, {I}_{L}^{P}(t)\right)$ is globally stable. The threshold condition ${\lambda }_{1}<1$ ensures the existence of a periodic solution $\left({E}^{P}\left(t\right), {I}_{L}^{P}(t)\right)$ and ${\lambda }_{2}<1$ guarantees the stability of the tumour-free periodic solution $\left({E}^{P}\left(t\right),0, {I}_{L}^{P}(t)\right)$, as shown in Fig. S.2e and f. However, if ${\lambda }_{2}>1$ then the tumour mass can periodically oscillate which can be detected or undetected with a significant variation period (Fig. S.2 g and h). The threshold conditions not only provide the relations of the parameters related to the interactions among effector cells, tumour cells and anti-tumour cytokines, but also reveal the relations between the dose and timing of combination therapy, which can help in the design of optimal dose and combination therapy so as to reduce the quantity of tumour cells and eradicate them eventually.

### Methods for exploring the hormetic effects

In order to reveal the RCRC and/or the IRC implied by the dynamics of model (1), we focus on two cases: 1) Monitoring the outcomes of combination therapy simultaneously, i.e. we monitor the number of effector cells (${E}_{n}\triangleq E(nP)$), tumour cells (${T}_{n}\triangleq T(nP)$) and cytokines (${I}_{Ln}\triangleq {I}_{L}(nP)$) at each time point $nP(n=\mathrm{0,1},2,\cdots )$, and then the combination therapy is applied simultaneously at $nP$ with updated values, denoted by ${E}_{n}^{+}, {T}_{n}^{+}, \mathrm{and} {I}_{Ln}^{+}$. Moreover, we can have the following relations:

2 $$\left\{\begin{array}{c}{E}_{n+1}^{+}={\left(1-{q}_{1}\right)\Phi }_{E}\left({E}_{n}^{+}, {T}_{n}^{+}, {I}_{Ln}^{+},\theta \right)+{s}_{1}, \\ {T}_{n+1}^{+}={\left(1-{q}_{2}\right)\Phi }_{T}\left({E}_{n}^{+}, {T}_{n}^{+}, {I}_{Ln}^{+},\theta \right), \\ {I}_{Ln+1}^{+}={\left(1-{q}_{3}\right)\Phi }_{I}\left({E}_{n}^{+}, {T}_{n}^{+}, {I}_{Ln}^{+},\theta \right)+{s}_{2},\end{array}\right.$$

where ${\Phi }_{E},{\Phi }_{T} \mathrm{and} {\Phi }_{I}$ are determined by the solutions of the ordinary differential equation (ODE) part of model (1) which can be integrated in the interval $(nP, (n+1)P]$, and $\theta$ denotes the parameter vector of all parameters in model (1). Thus, (2) is a Poincaré map or stroboscopic map of model (1), and the existence and stability of fixed points of (2) and their relations with the control parameters ${q}_{1},{q}_{2},{q}_{3, }{ s}_{1}\mathrm{and} {s}_{2}$ are crucial for the RCRC and/or the IRC. 2) Detection of the number of tumour cells and applying combination therapy are not carried out at the same time, i.e. the number of tumour cells is measured at checkpoints during the monitoring of a patient after diagnosis rather than at the treatment time, see details in Sect. 4 of Supplementary Material.

To realize the above purposes and to carry out one parameter or two-parameter bifurcation analyses, we chose two key parameters including an instant killing rate ${q}_{2}$, which directly relates to the dose of radiotherapy and/or chemotherapy (dose dependent parameter), and an administration constant ${s}_{1}$ which represents the immunotherapy. In order to reveal the influence of randomness on steady-state solutions and multiple attractors, we employed a uniform distribution to randomly generate initial values and to solve model (1) numerically until combination therapies have been implemented 200 times. Finally, the RCRC and/or the IRC were obtained, based on bifurcation parameters, by calculating the average value of the last 51 detection points. The influence of other parameters can be examined by parameter sensitivity analyses. The base line parameter values of model (1) were chosen from the literature, but for the convenience of calculations [7, 8], we have made some dimensionless changes, as discussed in the Supplementary Material. Meanwhile, in order to reveal the influence of system parameters on the dynamics of the RCRC and/or the IRC, two different sets of parameters are used throughout the paper.

### Hormetic data sets and data fitting

As far as we know, there are no published data on interactions between tumour cells, immune cells and effector cells in a combination therapy. Moreover, most of the data sets reported in the literature derive from single radio/chemotherapy or immunotherapy treatments, i.e. the published data sets are almost all RCRC curves under single doses of radio/chemotherapy, or the IRC curves under single doses of immunotherapy, with no published MRC curves at all. Therefore, there are no repeated experimental or monitoring data sets under multiple periodic radio/chemotherapy or immunotherapy available. So, we used the dose-related parameters of radio/chemotherapy or immunotherapy as the bifurcation parameters and employed the mean value of the stable state of the tumour cells in the proposed system to fit published RCRC or IRC curves [17, 33], to reveal the validity of the model in fitting the hormetic response curves of radio/chemotherapy or immunotherapy. By employing the least squares method, the data set in vitro assay system related to the hormetic dose response relationships of anti-cancer agents for lung cancer^33^ and the data set for the differences in tumour growth related to the immune reactant shown in Fig. 1d of reference (17) were used to fit the RCRC and IRC curves generated by our proposed model.

## Results

### One-parameter bifurcation diagrams for the RCRC

**Fig. 2:**
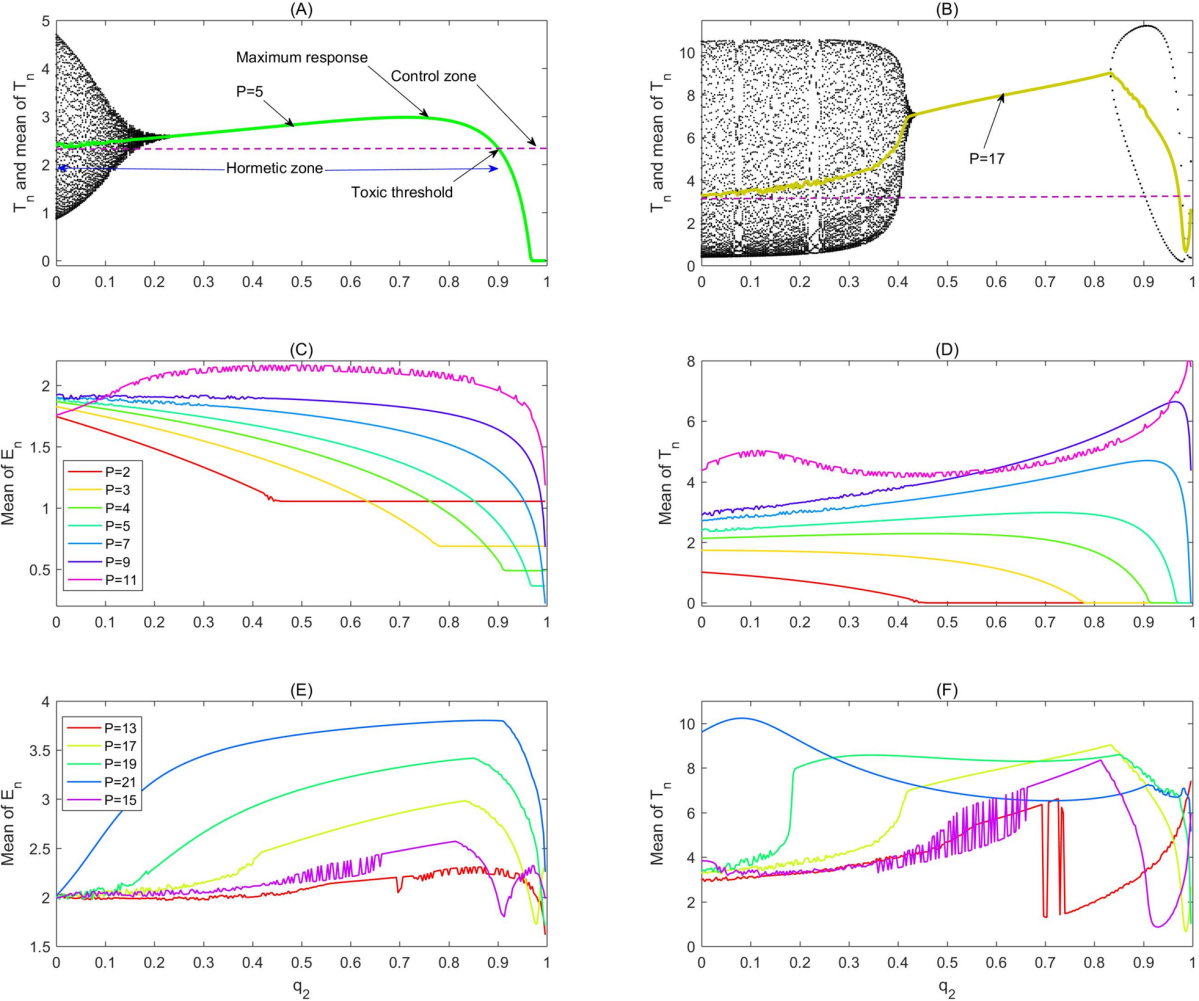
The hormetic effects of model (1). The baseline parameter values are as follows: $\mathrm{c}=0.08, {\mu }_{2}=0.1667, { p}_{1}=0.6917,{ g}_{1}=10, { g}_{2}=10, { r}_{2}=1,\mathrm{ b}=0.03,\mathrm{ a}=5.5556, {\mu }_{3}=55.556,{ p}_{2}=27.7778,{ g}_{3}=1,{ q}_{1}=0.08,{ s}_{1}=0.5, {q}_{3}=0.08, {s}_{2}=0.5$ with $\mathrm{P}$ and ${q}_{2}$ varying as shown in each subplot. **a**-**b** Bifurcation diagrams for model (1) with $P=5\mathrm{ and }17$; **c**-**f** RCRCs with period $P$ varying from 2 to 21

One parameter bifurcation analyses shown in Fig. 2a and b for two different administration periods $P$ depict how the ${T}_{\mathrm{n}}$ and its mean vary as the bifurcation parameter ${q}_{2}$ increases. Moreover, it reveals the formulation of the RCRC as the administration dose changes, in which the hormetic and control zones, toxic threshold and maximum response have been marked. Comparing (a) with (b), we could conclude that the period of combination therapy can significantly influence the characteristics of the RCRC. The RCRCs with respect to a wide range of period $\mathrm{P}$ are shown in Fig. 2 (c-f), which indicates that too frequent administration of the radio/chemotherapies (i.e. $P$= 2 and 3) cannot generate any hormetic effects provided that the side-effects of radio/chemotherapy on effector cells and cytokines are relatively low (here ${\mathrm{q}}_{1}=0.08,{\mathrm{q}}_{3}=0.08)$. However, the RCRC occurs once the administration period $P$ slightly increases, for example $P$= 5 and 6, as shown in Fig. 2a and d. The results in Fig. 2d reveal that the toxic thresholds are shifted from left to right by increasing the administration period $P$, enhancing the hormetic effects including a widened hormetic zone and increased maximum response. When the therapy period is increased further, the RCRC becomes very complex, even making an inverted U-shape and a U-shape appear successively as the instant killing rate ${q}_{2}$ increases, resulting in complex hormetic effects, for example with $P$= 15 and 17 as shown in Fig. 2b and f.

**Fig. 3:**
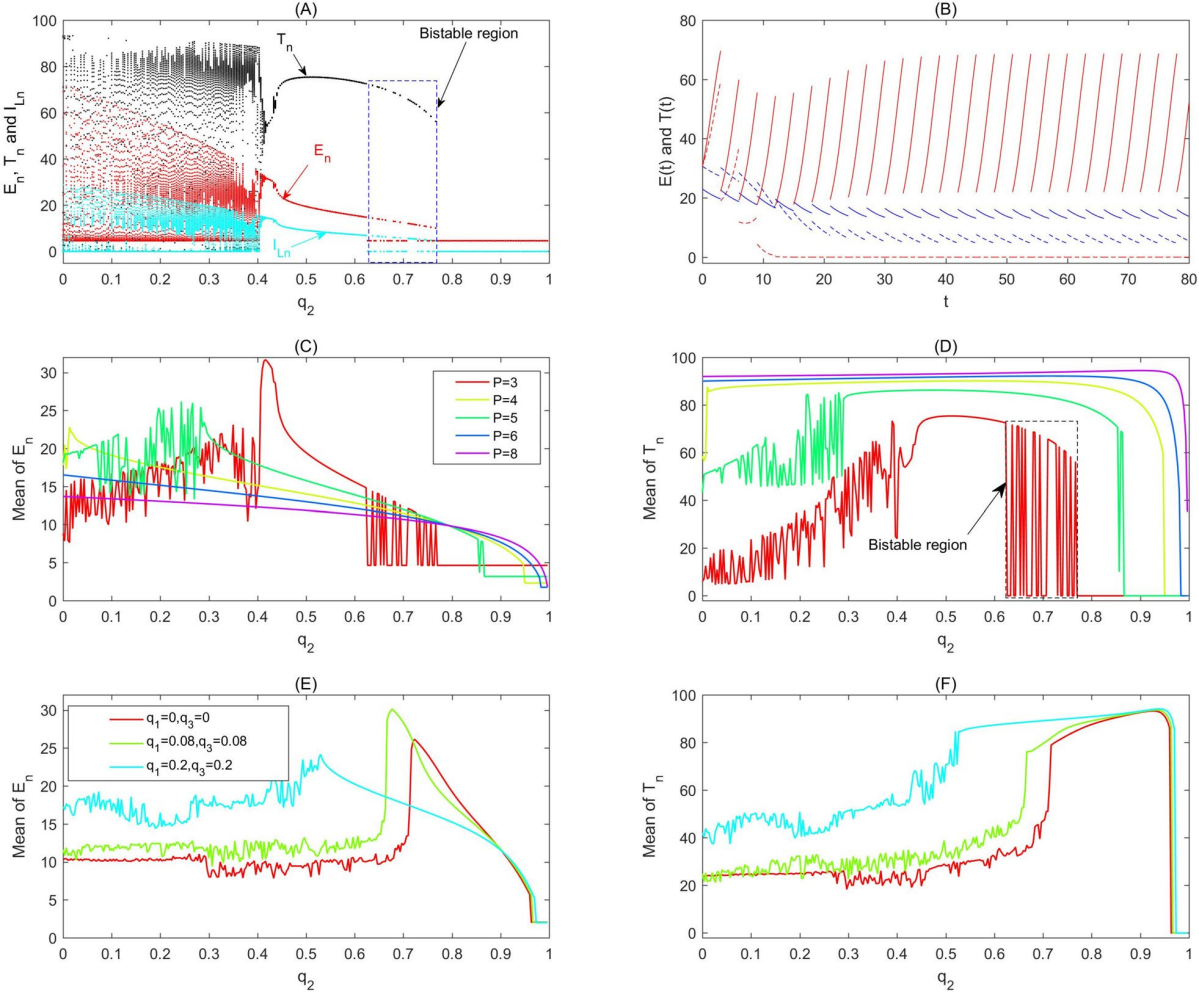
The hormetic effects of model (1). The baseline parameter values are as follows: $\mathrm{c}=0.0128, {\mu }_{2}=0.1667, { p}_{1}=0.6917,{ g}_{1}=70, { g}_{2}=5, { r}_{2}=1,\mathrm{ b}=0.01,\mathrm{ a}=0.55556, {\mu }_{3}=55.556,{ p}_{2}=27.7778,{ g}_{3}=5,{ q}_{1}=0,{ s}_{1}=3.5,{ q}_{2}=0.3, {q}_{3}=0, {s}_{2}=3$ with $P$ and ${q}_{2}$ varying as shown in each subplot. **a** Bifurcation diagrams for model (1) with $P=3$; **b** Two attractors coexist and bi-stability occurs for a wide range of parameters shown in (**a**) with $P=3$ and ${q}_{2}=0.68$; **c** and **d** The influences of period $P$ on the hormetic effect; **e** and **f** The influences of side-effects of ${q}_{1}$ and ${q}_{3}$ on the hormetic effects with $P=12,{s}_{1}={s}_{2}=13$, respectively

The low dose level stimulations can be evinced as a new equilibrium of stroboscopic map 2 (given in Methods). The tumour cells’ intrinsic reproductive capacity is not fully expressed under natural conditions, but a low dose radio/chemotherapy perturbation may result in hormetic effects such that the tumour size is pushed beyond its previous homeostatic state to a new larger equilibrium (called a homeostatic change [25]). Moreover, catastrophic shifts can also generate hormetic biphasic dose responses. To show this we chose the second parameter set shown in Fig. 3 and carried out similar bifurcation analyses. The bifurcation diagram for ${T}_{n}$ (Fig. 3a) shows that catastrophic shifts do occur for a wide range of doses ($\mathrm{the bistable region for }{q}_{2}$). The corresponding RCRC for ${T}_{n}$ is shown in Fig. 3d, marked in red. For example, the tumour free periodic solution and the interior periodic solution can coexist (red denotes the $\mathrm{T}(\mathrm{t})$ and blue represents the $\mathrm{E}(\mathrm{t})$), as shown in Fig. 3b. Figure 3c and d reveal that the hormetic zones are widened and that the maximum responses and toxic threshold increase significantly, and the bi-stable regions disappear quickly as the period $\mathrm{P}$ increases or varies, which indicate that the RCRC is very sensitive to the change of parameters under high frequency and low dose immunotherapy. The effect of low frequency and high dose immunotherapy on the RCRC is revealed in Fig. 3e and f, which show that the range of the hormetic zone is significantly increased and that the side-effects of radiotherapy and/or chemotherapy have a major influence on the shape of the RCRCs, yet have a very small influence on the toxic thresholds.

**Fig. 4:**
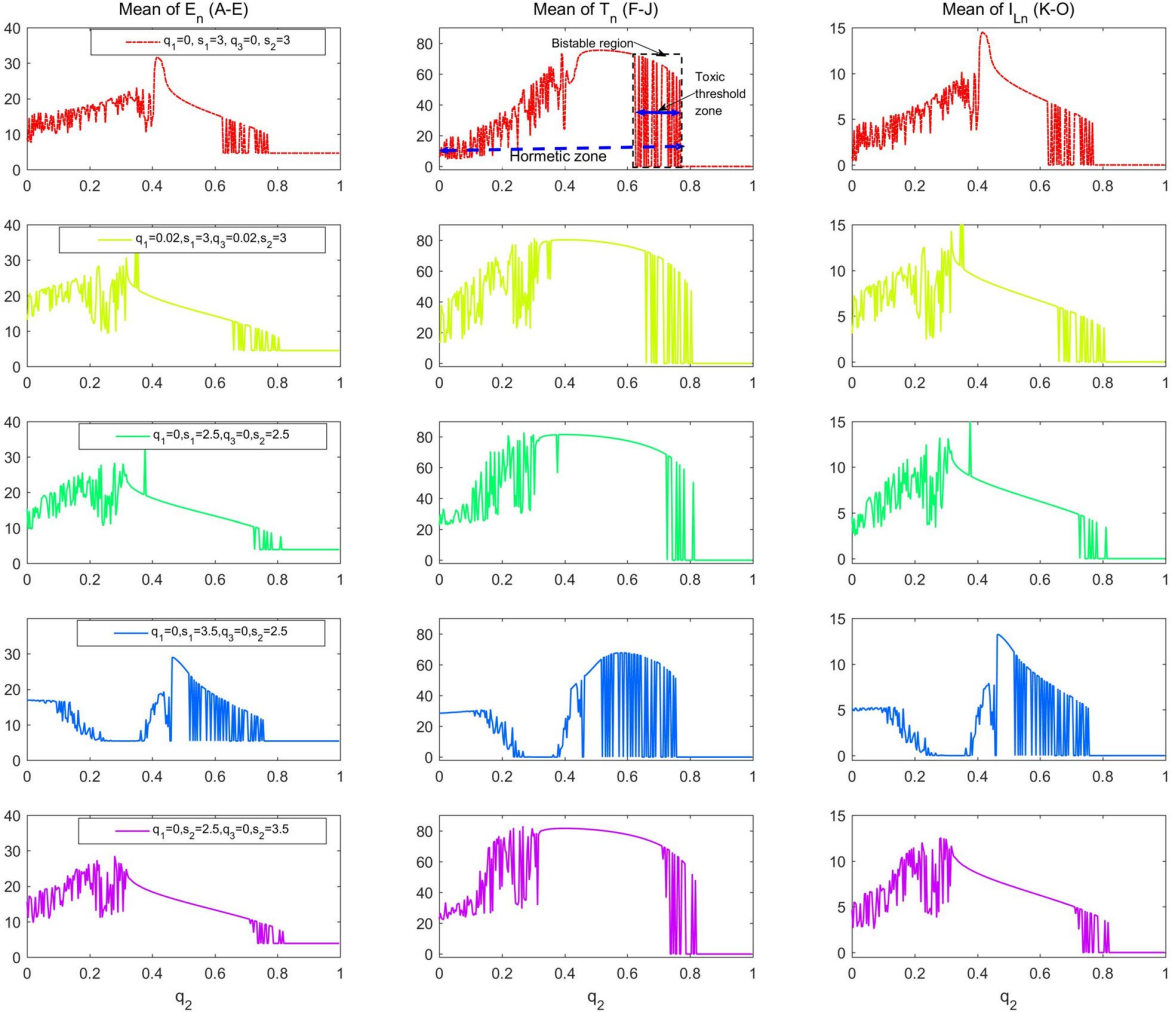
RCRC with radio/chemotherapy. Bifurcation diagrams with respect to killing rate ${q}_{2}$ to reveal the hormetic effects of model (1), with the values of ${E}_{n}, {T}_{n}, {I}_{Ln}$ calculated for $\mathrm{E}(\mathrm{t}),\mathrm{ T}(\mathrm{t})$ and ${I}_{L}(t)$. The baseline parameter values are as follows: $\mathrm{c}=0.0128, {\mu }_{2}=0.1667, { p}_{1}=0.6917,{ g}_{1}=70, { g}_{2}=5, { r}_{2}=1,\mathrm{ b}=0.01,\mathrm{ a}=0.55556, {\mu }_{3}=55.556,{ p}_{2}=27.7778,{ g}_{3}=5,{ q}_{1}=0,{ s}_{1}=3, {q}_{3}=0, {s}_{2}=3, P=3$. **a**-**e** The mean of ${E}_{n}$ over the last 51 impulsive stimulations; **f**-**j** The mean of ${T}_{n}$ over the last 51 impulsive stimulations; **k**–**o** The mean of ${I}_{Ln}$ over the last 51 impulsive stimulations. The four control parameters (${q}_{1}, {s}_{1}, {q}_{3}, {s}_{2}$) vary as shown in each subplot

Model (1) exists with bi-stable regions for a wide range of parameters, which correspond to the hormetic zone and toxic threshold. Thus, a bi-stable region poses new challenges in determining the hormetic zone and toxic threshold, which can change over a wide range of doses and depends strictly on the initial values of effector cells, tumour cells and cytokines. In particular, the existence of bi-stable regions causes the hormetic zone and toxic threshold to have a “fuzzy region”, that is, different clinical trial conditions or small randomness may lead to a large deviation in the monitored hormetic zones and toxic thresholds. Therefore, the bi-stable region can also be referred to as the “mixed fuzzy region” of the hormetic zone and toxic threshold. More detailed bifurcation analyses with respect to four key parameters ${(\mathrm{i}.\mathrm{e}. q}_{1}, {s}_{1}, {q}_{3}, {s}_{2})$ depict the effects of combination therapy on the “mixed fuzzy region” (Fig. 4). Among them, we mark two basic characteristics of the hormetic model including the hormetic zone and toxic threshold with “mixed fuzzy region” (Fig. 4f). The side-effects of radio/chemotherapy on basic characteristics of the RCRC are first shown in Fig. 4g, in which the parameter values ${q}_{1}\mathrm{ and }{q}_{3}$ are slightly increased. Obviously, the maximum response and toxic threshold are increased, and the hormetic zone or mixed fuzzy region expanded to the right. This indicates that small side-effects of radio/chemotherapy can have an important impact on the basic characteristics of the hormetic model. If we reduce the intensity of immunotherapy each time, i.e. reduce the values of both parameters ${s}_{1}\mathrm{ and }{s}_{2}$, Fig. 4h reveals that the RCRC has changed a lot, insofar as the width of the mixed fuzzy region is significantly narrowed.

When ${s}_{1}=3.5\mathrm{ and }{s}_{2}=2.5$, we find that U-shape and inverted U-shape RCRCs appear successively when the parameter ${q}_{2}$ increases (Fig. 4i and j). Under the immunotherapy strategy of strong application of effector cells, low-dose radio/chemotherapy combined with strong immunotherapy is very effective, and even clears tumour cells for a certain therapy regime. These results confirm that low-dose radio/chemotherapy is able to enhance immune functions, and then delay tumour progression [10–12, 20, 29]. This phenomenon of the beneficial effects of low-dose radiation (and/or low-dose metronomic chemotherapy) is often called ‘radiation hormesis’ [10–12, 20, 29]. Moreover, the main results shown in Fig. 4i indicate that low-dose radiation can be curative of cancer or at least delay its progression, leading to fewer cancer related deaths and no side-effects. With increasing doses of radio/chemotherapy, the inverted U-shape hormetic effect occurs, which again leads to a large increase of tumour cells. Moreover, the bi-stable region (mixed fuzzy region) is further expanded, making it more difficult to determine the toxic threshold. The results shown in Fig. 4i reveal that immunotherapy, through its effector cells, is more likely to be effective than the release of cytokines, in comparison with the results shown in Fig. 4j.

### IRC and MRC with radio/chemotherapy-immunotherapy

**Fig. 5:**
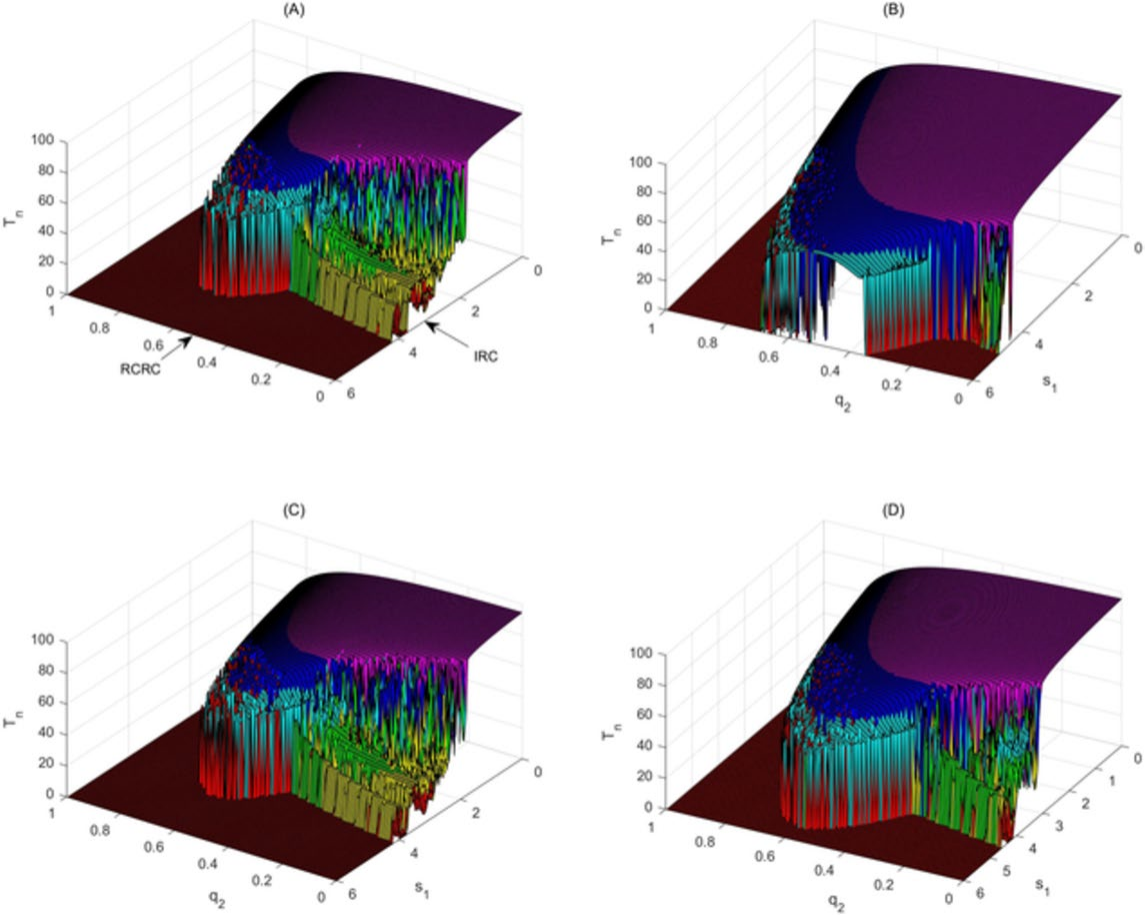
Mixed response curve (MRC) with radio/chemo-immunotherapy. Bi-parameter bifurcation diagrams with respect to killing rate ${q}_{2}$ and immune constant ${s}_{1}$ to reveal the immune and dose hormetic effects of model (1), with the values of ${E}_{n}, {T}_{n}, {I}_{Ln}$ calculated for $E(\mathrm{t}), T(\mathrm{t})$ and ${I}_{L}(t)$. The baseline parameter values are as follows:$\mathrm{c}=0.0128, {\mu }_{2}=0.1667, { p}_{1}=0.6917,{ g}_{1}=70, { g}_{2}=5, { r}_{2}=1,\mathrm{ b}=0.01,\mathrm{ a}=0.55556, {\mu }_{3}=55.556,{ p}_{2}=27.7778,{ g}_{3}=5,{ q}_{1}=0, {q}_{3}=0, {s}_{2}=2.5, P=3$ in (**a**), ${q}_{1}= {q}_{3}=0.1$ in (**b**), ${s}_{2}=0.5$ in (**c**), and ${q}_{1}= {q}_{3}=0.05$ in (**d**)

One-parameter bifurcation diagrams for RCRCs or IRCs reveal that dosages of radiation, chemotherapy and immunotherapy have important impacts on the occurrence of paradoxical and hormetic effects. To address this, we employed the two base line parameter sets mentioned above and chose the instant killing rate ${q}_{2}$ and the administration constant ${s}_{1}$ as bifurcation parameters. The two-parameter bifurcation diagrams shown in Fig. 5 show the basic characteristics of the MRC as parameters ${q}_{2}$ and ${\mathrm{s}}_{1}$ increase simultaneously, and the dynamic relationship between the RCRC and the IRC is revealed.

Intuitively, when radio/chemotherapy does not have side-effects on effector cells and cytokines, high-dose radio/chemotherapy and strong immunotherapy are conducive to the inhibition or even eradication of tumour cells (Fig. 5a). For a given effector cell administration constant (fixed ${s}_{1}$ here), there still exists a dose region of radio/chemotherapy (a range of ${q}_{2}$ here), which causes occurrence of the hormetic effect and appearance of the RCRC. Similarly, for a given dose of radio/chemotherapy (fixed ${q}_{2}$ here), there also exists an effector cell administration region, which makes the IRC appear. However, once the side-effects of radio/chemotherapy on effector cells and cytokines have been accounted for, the MRCs are significantly affected, as shown in Fig. 5b and d. The phenomenon revealed in Fig. 5b is the existence of a strong immunotherapy region in both low-dose and high-dose radiation/chemotherapy regions. Moreover, in both regions, tumour cells can be effectively eradicated. As mentioned above, in contrast to high-dose radiation/chemotherapy, low-dose radiation/chemotherapy has many advantages in tumour treatment, such as strategies that are easy to design and optimize, not too many side-effects, etc. The conclusions revealed by Fig. 5 are of major importance for establishing the optimal scheduling of immunology and chemotherapy, to identify whether this effect is limited to a specific type of radio/chemotherapy and whether immunotherapy can or cannot also augment the clinical effect of radio/chemotherapy. The effect of reducing the administration constant ${\mathrm{s}}_{2}$ of cytokines on MRCs is not obvious (Fig. 5c), which further explains the importance of the design of optimal immunotherapy. It is worth noting that immunotherapies that potentially stimulate tumour growth (IRC occurs here) may at the same time sensitize a tumour to low-dose radio/chemotherapy, and therefore the net effect would still be beneficial.

**Fig. 6:**
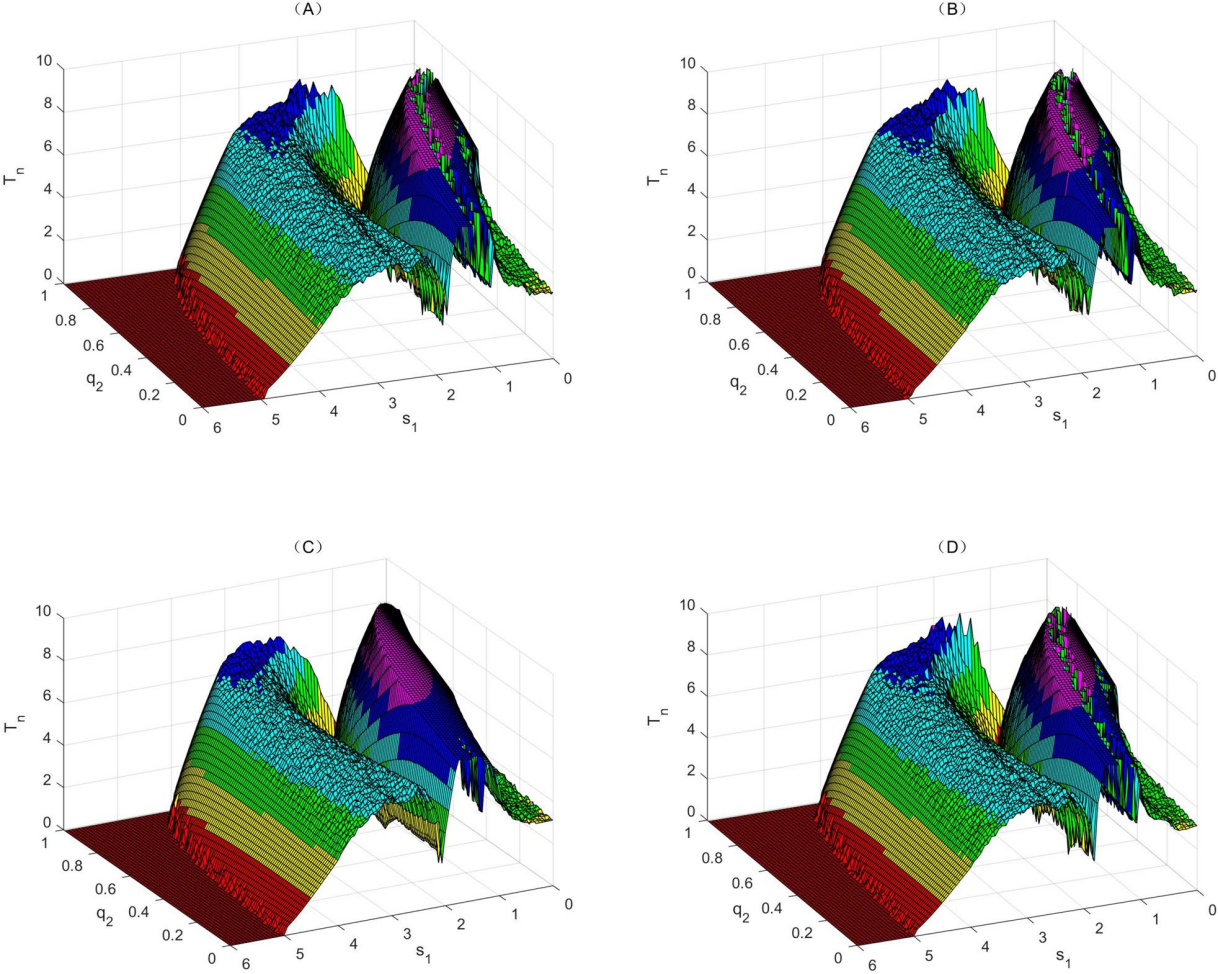
Multi-hump MRC for radio/chemo-immunotherapy. Bi-parameter bifurcation diagrams with respect to killing rate ${q}_{2}$ and immune constant ${s}_{1}$ to reveal the immune and dose hormetic effects of model (1), with the value of ${T}_{n}$ calculated for $\mathrm{T}(\mathrm{t})$. The baseline parameter values are as follows:$\mathrm{c}=0.08, {\mu }_{2}=0.1667, { p}_{1}=0.6917,{ g}_{1}=10, { g}_{2}=10, { r}_{2}=1,\mathrm{ b}=0.03,\mathrm{ a}=5.5556, {\mu }_{3}=55.556,{ p}_{2}=27.7778,{ g}_{3}=1,{ q}_{1}=0.08,{ s}_{1}=0, {q}_{3}=0, {s}_{2}=0, P=17$ in (**a**), ${q}_{3}=-0.1$ in (**b**), ${{q}_{1}=0.1,q}_{3}=-0.1$ in (**c**), and ${q}_{3}=-0.3$ in (**d**)

The dual effects of radio/chemotherapy on cytokines show that the combined treatment can inhibit cytokine growth during treatment for some tumours, while stimulating their growth during the treatment for some other tumours [30, 31]. Therefore, the parameter ${q}_{3}$ may be positive or negative, and more importantly, it may be positive and negative alternately, depending on the experimental design. Therefore, in the following we chose the second parameter set and carried out two-parameter bifurcation analyses for various values of ${q}_{3}$, as shown in Fig. 6, from which we can see that multiple inverted U-shaped RCRCs and IRCs are duplicated (which we denote multi-hump MRCs).

For a fixed intensity of radio/chemotherapy (including ${q}_{2}=0$ in Fig. 6a), we first consider the influence of dynamic changes in the immunotherapy regime on tumour cells, i.e. for a given ${q}_{2}$, the administration constant ${\mathrm{s}}_{1}$ of effector cells is increased from 0 to 6, resulting in an IRC with multiple humps. The mean value of ${T}_{n}$ increases rapidly with increasing administration of effector cells, reaching a peak, followed by a second peak after a sudden decline and rapid rise process. After another slow decline and rise process, it reaches a new peak, and finally slowly declines until the tumour cells can be cleared for a large enough administration constant. The IRCs can be significantly influenced by increasing the intensity of radio/chemotherapy (increasing ${q}_{2}$ here). In conclusion, the stronger the radio/chemotherapy, the more rapid the occurrence of the IRC. The positive or negative effects of radio/chemotherapy on cytokines are considered in Fig. 6b-d and, in general, external perturbations of cytokines have little effect on IRCs. However, the changes in effector cells do have a significant effect on RCRCs, which mainly depends on the internal mechanism of interactions among tumour cells, effector cells and cytokines. Therefore, the MRC can help us to understand this complex relationship and provide a decision-making basis for the design of an optimal combination therapy.

### The effects of checkpoint and treatment period on the dynamics including those of the RCRC

Usually the number of tumour cells is measured at checkpoints when the patient’s cancer was diagnosed and at subsequent follow-ups, rather than being measured at the treatment point. However, a question arises about how do the numbers of tumour cells measured at checkpoints during the monitoring of patients after diagnosis, rather than at the treatment time, influence the dynamics including those of the RCRCs and IRCs? Theoretical analyses shown in Sect. 4 of the Supplementary Material reveal that the treatment time point does not affect the existence and stability of the tumour free periodic solution if we fix the therapy period. However, the difference between the treatment time and the monitoring time makes the evolving curve of tumour cells and the main characteristics of the hormetic model (Fig. 1) change significantly, as shown in Figs.S.2, S.3 and S.4, explained in Sect. 4 of the Supplementary Material.

In particular, one parameter bifurcation diagrams reveal that the numbers of tumour cells in the monitoring site and the treatment site are very different(shown in Fig.S.5 of the Supplementary Material): the maximum responses are essentially different, and the mixed fuzzy regions also change slightly, as shown in Fig. S.4. This indicates that the RCRCs can be significantly affected if we record the number of tumour cells at different time points. However, the values of ${T}_{n}$ (i.e. measures of the number of tumour cells before the treatment) do not change too much as the treatment period, $P$, changes. Nevertheless, the two-parameter bifurcation diagrams shown in Fig.S.6 of the Supplementary Material reveal that the numbers of tumour cells or MRCs detected at two different time points may change significantly with the change of parameters. There is no doubt that the huge difference in monitoring or detecting tumour cells at different times will have a major impact on the treatment plan, and then on the treatment effect. Therefore, it is very important to design a reasonable treatment and detection scheme.

### Heterogeneous effects of radio/chemotherapy on tumour growth

Various anti-tumour cytokines and experimental designs could result in positive or negative signs of parameter ${q}_{3}$. Thus, we consider a more generalized case here i.e. let ${q}_{1},{q}_{3}$ be generated from an uniform distribution at each time $nP$, i.e. ${q}_{1}\in U\left(-\mathrm{0.2,0.2}\right)$ and ${q}_{3}\in U\left(-\mathrm{0.2,0.2}\right)$, and discuss how the stochasticity of experimental designs can influence the evolution of heterogeneous tumour cells [30, 31], achieved by employing the methods and numerical techniques proposed in reference [32].

**Fig. 7:**
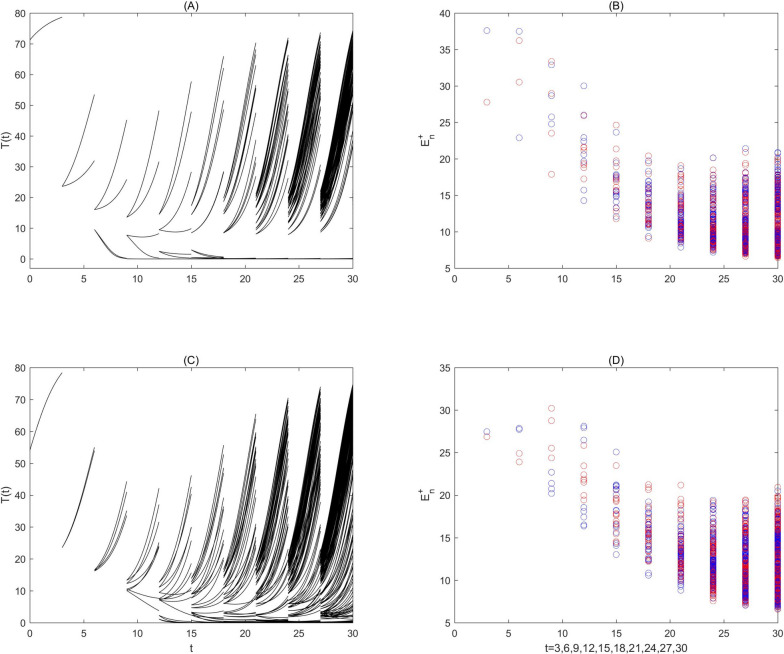
Heterogeneous effects of radio/chemotherapy on tumour growth. Two independent simulations (a and b; c and d) with random initial values and random perturbation parameters ${q}_{1}\in U\left(-\mathrm{0.2,0.2}\right)$ and ${q}_{3}\in U\left(-\mathrm{0.2,0.2}\right)$. The baseline parameter values are as follows:$\mathrm{c}=0.0128, {\mu }_{2}=0.1667, { p}_{1}=0.6917,{ g}_{1}=70, { g}_{2}=5, { r}_{2}=1,\mathrm{ b}=0.01,\mathrm{ a}=0.55556, {\mu }_{3}=55.556,{ p}_{2}=27.7778,{ g}_{3}=5,{ {s}_{1}=3, q}_{2}=0.7, {s}_{2}=3, P=$ 3. The evolutions of $T(\mathrm{t})$ and ${E}_{n}^{+}$ are shown in (**a**) and (**c**) and (**b**) and (**d**), respectively

Two independent simulations with random initial values and random generated parameters ${q}_{1}$ and ${q}_{3}$ are shown in Fig. 7 with the parameter set fixed in the mixed fuzzy region, which represents the evolution of the number of tumour cells for$n=\mathrm{1,2},\cdots ,10$. At each time point$nP$, two coefficients ${q}_{1},{q}_{3}$ have been independently drawn and describe the heterogeneous effects, which result in two different values of${E}_{n}^{+} \mathrm{and} {I}_{Ln}^{+}$, and two values for ${E}_{n}^{+}$ are shown in Fig. 7b and d marked in red and blue $.$ After a few combination therapies, a low-numbered pool of tumour cells emerges associated with tumour-free solutions, and a high numbered pool of tumour cells, associated with tumour growth with a total number of ${2}^{\mathrm{n}-1}$ trajectories for $T(\mathrm{t})$ in each time interval $\left[\left(\mathrm{n}-1\right)\mathrm{P},\mathrm{ nP}\right]$ (Fig. 7a and c). The number of tumour cells showed two different characteristics with the increase of *n* in two independent random simulations. The final fates of each complete trajectory corresponding to the number of tumour cells is very clear in Fig. 7a after several rounds of combination therapies. However, the final fates of some trajectories shown in Fig. 7c are undetermined. Clearly, variation in anti-tumour cytokines and heterogeneous effects of radio/chemotherapy on tumour growth are complex.

### Model fitting to clinical data

**Fig. 8:**
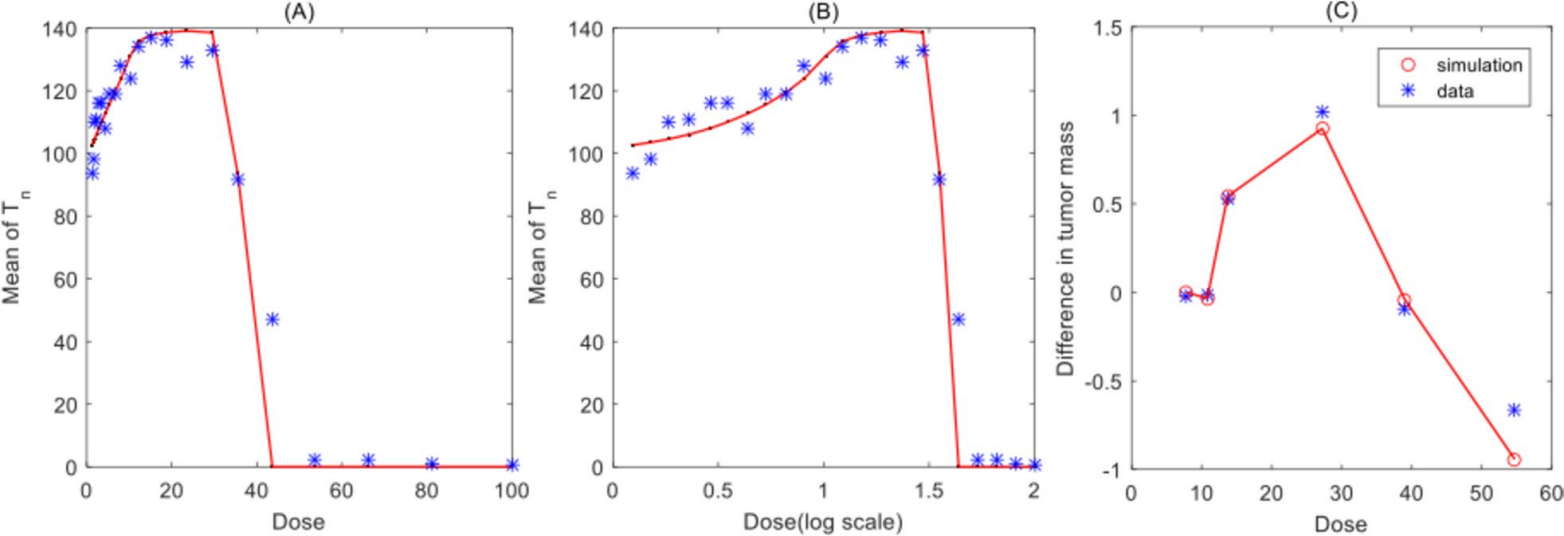
Fitting of RCRC and IRC hormetic data sets from references [31] and [17], respectively. Red lines show the simulation results and blue stars show the data. **a**-**b** Hormetic radio/chemotherapy dose response curves with different dose scales; **c** Immune response curve

As emphasized in the methods section, we employed the mean value of stable states of the tumour cells in system (1) to fit the RCRC or IRC data sets shown in Fig. 8 [17, 33]. We define $\mathrm{D}$ as the radio/chemotherapy or immunotherapy dose and consider the control parameters to be dose dependent. Thus, the parameter ${q}_{2}$ is chosen to be the bifurcation parameter related to the hormetic radio/chemotherapy dose response and the parameter ${\mathrm{s}}_{1}$ is chosen to be the bifurcation parameter related to the immune reactant. The relationships between the two parameters and the radio/chemotherapy or immunotherapy dose are ${q}_{2}=1-{e}^{-{r}_{q}D}$ and ${\mathrm{s}}_{1}={p}_{s}D$. Then the least squares method was used to estimate the unknown parameters of the model based on the two RCRC and IRC hormetic data sets, respectively. The estimation results are shown in Table S.1 in Supplementary Material and Fig. 8, which not only reveal that a low radio/chemotherapy dose or a low quantity of immune reactants stimulate tumour growth, but also demonstrate that the proposed model fits the various real data sets well.

## Discussion

Although the modes of action and synergies involved in administering immunotherapy with radiotherapy or chemotherapy are unexplained, it is however clear that such combinations are effective against a variety of cancers [1, 4–6]. Here we used the classic dynamic model of interactions between tumour cells, effector cells and cytokines, to address several of the possible mechanisms involved. For this aim, we explored the combined effect of pulsed immunotherapy and radio/chemotherapy on tumours, with especial regard to the conditions and mechanisms leading to paradoxical and hormetic effects in relation to different treatment schemes, through bifurcation analyses based on key parameters. By using published parameter sets and making appropriate changes to increase the visibility of the figures in this paper, numerical bifurcation analysis techniques were used to reveal the parameter space and the mechanisms underlying paradoxical and hormetic effects produced by radio/chemotherapy, immunotherapy and combination therapy.

The mechanisms involved in this synergy and the clinical implications of RCRCs and/or IRCs of cancer are not yet clear, but there are potential considerations that could either benefit or harm cancer patients. The model revealed that the RCRC and/or the IRC or a combination of the two, termed a mixed response curve (MRC), undergo homeostatic changes or catastrophic shifts and have hormetic effects in many parameter regions. Some of the MRCs have multiple humps which pose challenges for the interpretation of clinical trials and for experimental design, due to a fuzzy region between an hormetic zone and the toxic threshold. MRCs generated by two parameter bifurcation analyses based on radio/chemotherapy and immunotherapy revealed that low-dose radio/chemotherapy and strong immunotherapy can counteract the side-effects of radio/chemotherapy on effector cells and cytokines, as well as stimulating effects of immunotherapy on tumour cell growth. Thus, the net benefit and synergistic effect of combined therapy is conducive to the treatment and inhibition of tumour cells and the implications for potential clinical applications were confirmed by fitting our model to RCRC and IRC data sets.

The conclusions of the mathematical model and numerical analysis method developed here are consistent with those revealed by many experiments [10–12, 20], which shows that the combination of low-dose radio/chemotherapy and high-dose immunotherapy is very effective for many solid tumours, lung cancer or gastric cancer. Furthermore, the numerical investigation also revealed the important influence of the change of detection point and treatment starting point on the number of tumour cells, and explains the importance of synchronous or asynchronous detection and treatment points as well as the synergistic effect on tumour treatment, which provides a basis for more in-depth theoretical and model analyses.

Whilst the conclusions from our model are optimistic insofar as they show that combination therapy with appropriate parameter values can have net benefits rather than fail, there are some caveats. One of these is the danger of cytokine release syndrome, potentially causing fever and organ failures [34], that can be a side-effect of excessive immune system stimulation [3]. Moreover, variation in anti-tumour cytokines and heterogeneous effects of radio/chemotherapy on evolution of tumour cells are complex, as shown in Fig. 7. This suggests that regular monitoring of tumour cell changes and heterogeneity may be crucial in the whole process of tumour treatment and confirms that precision medicine and individual based treatment are very important for tumour therapy.

Low dose radio/chemotherapy has dual effects in the regulation of immune hormesis (Fig. 1), but to further substantiate the effects that we have described clinical trials with appropriate design and statistical analysis are needed before protocols optimizing the immune response can be formalized. A number of clinical trials highlight major unresolved questions concerning the optimum choice, dosing, and timing of radio/chemotherapy relative to active immunotherapy [1, 4, 35]. In recent years, numerous studies have designed different combinations of radio/chemotherapy and immunotherapy in stage III cancers, evaluated the synergistic effects between radiotherapy or chemotherapy and immunotherapy, and have been approved and widely applied [36–39]. How to transform a small-scale randomized clinical experiment into a more determined optimized treatment strategy requires formulating and extending the mathematical model proposed in this article based on the above clinical trials. Furthermore, with the help of models and data analyses, we could provide the important qualitative and quantitative information for designing more accurate combination therapy plans, which can help us to achieve the maximum synergistic effect of combination therapy and reduce the probability of paradoxical and hormetic effects.

As the initial paper to study all kinds of treatment-induced paradoxical and hormetic effects by using the kinetic model, the limitation of this paper is that we only considered the key role played by dose and synergistic effects, and did not systematically study the effect of timing of radio/chemotherapy relative to active immunotherapy on the RCRC, IRC and MRC. Moreover, the model results have not been tested by pre-clinical experimental cell culture or animal models. However, experiments have shown that there is a delay between the initiation of chemotherapy and its immunosuppressive properties becoming evident [1, 3, 4, 35]. Thus, there is a period that provides a window of opportunity long enough to permit chemotherapy, combined with cytotoxic T-lymphocytes induced by the immunotherapy, to be effective against tumours. Therefore, the key questions are: when to start radiotherapy and chemotherapy after the administration of immunotherapy, and when to administer immunotherapy and what is the proper dosage for each of therapy after the end of radiotherapy and chemotherapy to maximize the synergistic effect of combined treatment? It would be important, in future research, to develop the dynamical model of the interaction among tumour cells, immune cells and effector cells for different stages of cancer patients based on clinical trails [36–39], and consider multi-pulse control sequences with different combination strategies to reveal what is the proper dosage for each kind of therapy to achieve maximum benefit. Taking advantage of results from mathematical models could help to further address the influence of the timing of radio/chemotherapy and immunotherapy on the synergistic effect of hormetic effects and maximize the synergistic effect of combination treatments.

### Supplementary Information

**Additional file 1:** **Supplementary Material.** Hormesis and synergistic e_ects of cancer treatments revealed by modeling combinations of radio- or chemotherapy with immunotherapy.

## Data Availability

Parameter estimates and other data are available in the Supplementary Material available with the online version of this article.

## Abbreviations

IRCs: Immune response curves
RCRCs: Radio/chemotherapy (dose) response curves
MRCs: Mixed response curves
