# Supplementary material for: Hormetic and synergistic effects of cancer treatments revealed by modelling combinations of radio - or chemotherapy with immunotherapy
Source: BMC Cancer. 2023 Oct 27;23:1040. doi: 10.1186/s12885-023-11542-6 (PMC10605942; doi:10.1186/s12885-023-11542-6)
Supplement: Supplementary file 1 — Additional file 1: Supplementary Material. Hormesis and synergistic e_ects of cancer treatments revealed by modeling combinations of radio- or chemotherapy with immunotherapy. [file 12885_2023_11542_MOESM1_ESM.pdf]

# Supplementary Material: Hormesis and synergistic effects of cancer treatments revealed by modeling combinations of radio- or chemotherapy with immunotherapy

## 1. The model

Our model describing the interaction among the effector cells, tumor cells, and the cytokine (IL-2) is as follows[1–8]:

$$\begin{cases} \left.\begin{aligned} \frac{dE(t)}{dt} &= cT - \mu_2 E + \frac{p_1 E I_L}{g_1 + I_L}, \\ \frac{dT(t)}{dt} &= r_2(T)T - \frac{aET}{g_2 + T}, \\ \frac{dI_L(t)}{dt} &= \frac{p_2 ET}{g_3 + T} - \mu_3 I_L, \end{aligned}\right\} & t \neq nP, \\ \left.\begin{aligned} E(t^+) &= (1 - q_1)E(t) + s_1, \\ T(t^+) &= (1 - q_2)T(t), \\ I_L(t^+) &= (1 - q_3)I_L(t) + s_2, \end{aligned}\right\} & t = nP, \end{cases} \tag{S1.1}$$

where $E(t)$ denotes the effector cells, which can activate immune-system cells such as cytotoxic T-cells, macrophages, and natural killer cells that are cytotoxic to the tumor cells. $T(t)$ represents the tumor cells, and $I_L(t)$ denotes the concentration of cytokines including IL-2 in the single tumor-site compartment. $q_2$ represents the tumor reduction rate in patients by exposure to radiotherapy and/or chemotherapy, and $q_1$ and $q_3$ depict side-effects of radiotherapy and/or chemotherapy on effector cells and the cytokine IL-2, respectively. Lastly, $s_1$ is a treatment term that represents an external source of effector cells such as LAK or TIL cells, and $s_2$ is a treatment term that represents an external input of cytokines into the system. The intrinsic growth rate of the tumor cells can be modelled by a linear growth term (in this case $r_2(T)$ is constant) or as a type of limited-growth with a logistic or Gompertz type function. Here we chose the

logistic growth function as follows:

$$r_2(t) = r_2(1 - bT).$$

The dynamical evolution of the effector cell population is described by the first equation, where $c$ represents the antigenicity of the tumor and $\mu_2$ denotes the death rate of the effector cell population, the Michaelis-Menten term (i.e. the third term) reveals the saturated effects of an immune response stimulated by cytokines with a Michaelis-Menten constant $g_1$ and maximal response constant $p_1$. The growth rate of the tumor cell population is given by the second equation with an intrinsic growth rate $r_2$ and a carrying capacity parameter b, the second term also shows the saturated effects of an immune response on the tumor cells stimulated by effector cells with two constants $g_2$ and $a$. The changing rate of the cytokine concentration is given by the third equation, which shows the effects of interactions between effector cells and tumor cells on the growth of cytokines through Michaelis-Menten kinetics with two constants $g_3$ and $p_2$, and $\mu_3$ denotes the degradation rate of the cytokines. See reference (1) for explanations of all of the parameters in more detail. The second part, consisting of three impulsive maps, describes the effects of pulses of radio/chemo-immunotherapy applied at period $P(n = 1, 2, \cdots)$ on the effector cells, tumor cells and IL-2. $E(t^+), T(t^+)$ and $I_L(t^+)$ denote the values after the pulsed therapy at time $t$, where $s_1$ is an administration constant that represents an external source of effector cells each time; $s_2$ is an administration constant that represents an external input of IL-2 into the system each time, $q_2$ represents the tumor reduction rate in patients by combined exposure to radio/chemotherapy, and $q_1$ and $q_3$ depict side-effects of radio/chemotherapy on the effector cells and the IL-2 with $0 \leq q_1, q_2 < 1$ and $q_3 < 1$.

Note that the anti-tumor cytokines including IL-2 and IL-12 in cancer diseases have been measured in different cancer types, and the levels of IL-12 in sera were found to be increased in metastatic patients and decreased in gastrointestinal tumours, colon cancer and breast cancer after radio/chemotherapy[9,10]. Those results indicate that radiotherapy and chemotherapy have dual effects

on cytokines, i.e. in some cases they inhibit the growth of cytokines, and in other cases stimulate the growth of cytokines. Therefore, $q_3$ may be positive or negative and more generalized cases are considered in the main text to show the effects of random perturbations on the dynamics of model (S1.1). Moreover, $0 < q_3 < 1$ indicates the reduction of levels of anti-tumor cytokines and $q_3 < 0$ means that the levels are increasing.

## 2. Existence and global stability of the tumor free periodic solution

The existence and stability of the tumor free periodic solution have been addressed in several publications[7,8], but their authors only focused on the local stability of some special cases. So, in the following we present general results for the existence and stability of the tumor free periodic solution. In order to calculate the tumor free periodic solution for system (S1.1), we first let $T = 0$, and obtain the following subsystem

$$\begin{cases} \left.\begin{aligned} \frac{dE(t)}{dt} &= -\mu_2 E(t) + \frac{p_1 E(t) I_L(t)}{g_1 + I_L(t)}, \\ \frac{dI_L(t)}{dt} &= -\mu_3 I_L(t), \end{aligned}\right\} \quad t \neq nP, \\ \left.\begin{aligned} E(t^+) &= (1-q_1)E(t) + s_1, \\ I_L(t^+) &= (1-q_3)I_L(t) + s_2, \end{aligned}\right\} \quad t = nP. \end{cases} \tag{S2.2}$$

Integrating the second equation $\frac{dI_L(t)}{dt} = -\mu_3 I_L$ within the interval $(nP, (n+1)P]$, gives

$$I_L(t) = \exp\left(-\mu_3(t - nP)\right) I_L(nP^+).$$

Thus, we have

$$I_L((n+1)P^+) = (1-q_3)\exp\left(-\mu_3 P\right) I_L(nP^+) + s_2.$$

Let $I_L^*$ be an equilibrium of the above equation, yielding

$$I_L^* = (1-q_3)\exp\left(-\mu_3 P\right) I_L^* + s_2$$

and solving it with respect to $I_L^*$, we get

$$I_L^* = \frac{s_2}{1-(1-q_3)\exp\left[-\mu_3 P\right]}.$$

Therefore, there exists a unique periodic solution for $I_L$, denoted by $I_L^P(t)$ with

$$I_L^P(t) = \exp\left[-\mu_3(t-nP)\right] I_L^*, \ t \in (nP,(n+1)P]. \tag{S2.3}$$

Then, substituting $I_L^P$ into the first equation of model (S2.2), one has the following equation

$$\frac{dE(t)}{dt} = -\mu_2 E(t) + \frac{p_1 E(t) I_L^P(t)}{g_1 + I_L^P(t)}, \ t \in (nP,(n+1)P].$$

Further, integrating it within the interval $(nP,(n+1)P]$, there is

$$E(t) = E(nP^+)\exp\left[-\mu_2(t-nP) + \int_{nP}^{t} \frac{p_1 I_L^P(t)}{g_1 + I_L^P(t)} dt\right].$$

Then through some straightforward calculation we have

$$\int_{nP}^{t} \frac{p_1 I_L^P(t)}{g_1 + I_L^P(t)} dt = -\frac{p_1}{\mu_3} \ln\left(\frac{g_1 + I_L^* e^{-\mu_3(t-nP)}}{g_1 + I_L^*}\right),$$

which indicates that

$$E((n+1)P^+) = (1-q_1)E(nP^+)\exp\left[-\mu_2 P - \frac{p_1}{\mu_3}\ln\left(\frac{g_1 + I_L^* e^{-\mu_3 P}}{g_1 + I_L^*}\right)\right] + s_1.$$

Consequently, we have

$$E^* = (1-q_1)E^* \exp\left[-\mu_2 P - \frac{p_1}{\mu_3}\ln\left(\frac{g_1 + I_L^* e^{-\mu_3 P}}{g_1 + I_L^*}\right)\right] + s_1$$

i.e.

$$E^* = \frac{s_1}{1-(1-q_1)\exp\left[-\mu_2 P - \frac{p_1}{\mu_3}\ln\left(\frac{g_1 + I_L^* e^{-\mu_3 P}}{g_1 + I_L^*}\right)\right]}.$$

Note that, to ensure the existence of $E^*$, we need

$$-\mu_2 P - \frac{p_1}{\mu_3}\ln\left(\frac{g_1 + I_L^* e^{-\mu_3 P}}{g_1 + I_L^*}\right) < \ln\frac{1}{1-q_1}. \tag{S2.4}$$

As a conclusion, if condition (S2.4) holds true, then subsystem (S2.2) has a unique periodic solution with period $P$, denoted by $(E^P(t), I_L^P(t))$. Here

$$E^P(t) = E^* \exp\left[-\mu_2(t-nP) - \frac{p_1}{\mu_3}\ln\left(\frac{g_1 + I_L^* e^{-\mu_3(t-nP)}}{g_1 + I_L^*}\right)\right], \ t \in (nP,(n+1)P].$$

Therefore, we have the following proposition.

**Proposition 1.** If condition (S2.4) holds true, then subsystem (S2.2) has a unique periodic solution $(E^P(t), I_L^P(t))$ with period $P$, which is globally stable.

*Proof.* As the existence and uniqueness have been discussed above, here we only prove the global stability of the periodic solution $(E^P(t), I_L^P(t))$. Firstly, we conduct the local stability analysis, which is determined by the two Floquet multipliers of the following matrix

$$M = \begin{pmatrix} 1-q_1 & 0 \\ 0 & 1-q_3 \end{pmatrix} \Phi(P),$$

where $\Phi(P)$ is a solution of the following equation at time point $P$

$$\frac{d\Phi(t)}{dt} = \begin{pmatrix} -\mu_2 + \frac{p_1 I_L^P(t)}{g_1 + I_L^P(t)} & * \\ 0 & -\mu_3 \end{pmatrix} \Phi(t)$$

with $\Phi(0) = I$ (the identity matrix). Here, $*$ indicates that the term is not necessary for the exact expression.

We can calculate the two Floquet multipliers of matrix $M$ as follows:

$$\lambda_3 = (1-q_3)e^{-\mu_3 P} < 1,\ \lambda_1 = (1-q_1)\exp\left[-\mu_2 P - \frac{p_1}{\mu_3}\ln\left(\frac{g_1 + I_L^* e^{-\mu_3 P}}{g_1 + I_L^*}\right)\right].$$

It is easy to find that $\lambda_1 < 1$ holds true in the condition (S2.4). This means that the periodic solution $(E^P(t), I_L^P(t))$ is locally stable whenever it exists. As for the global stability, we can easily see that the component $I_L^P(t)$ is globally stable and then by the theory of limitation systems we have the component $E^P(t)$ being also globally stable. This completes the proof.

Based on the above discussion, we know that model (S1.1) has a tumor free periodic solution (TFPS) $(E^P(t), 0, I_L^P(t))$ in the condition (S2.4). Next, we determine the threshold condition for the stability of the tumor free periodic solution, which could be useful for designing a combination therapy strategy.

Similarly, the local stability of the TFPS is determined by the Floquet mul-

tipliers of the following matrix

$$M_1 = \begin{pmatrix} 1-q_1 & 0 & 0 \\ 0 & 1-q_2 & 0 \\ 0 & 0 & 1-q_3 \end{pmatrix} \Phi(P),$$

and $\Phi(P)$ is a solution of the following equation at time point $P$

$$\frac{d\Phi(t)}{dt} = \begin{pmatrix} -\mu_2 + \frac{p_1 I_L^P(t)}{g_1+I_L^P(t)} & c & * \\ 0 & r_2(0) - \frac{aE^P(t)}{g_2} & 0 \\ 0 & \frac{p_2 E^P(t)}{g_3} & -\mu_3 \end{pmatrix} \Phi(t)$$

with $\Phi(0) = I$ (the identity matrix).

Through some straightforward calculations, the three Floquet multipliers of matrix $M$ are as follows:

$$\lambda_3 = (1-q_3)e^{-\mu_3 P} < 1, \ \lambda_1 = (1-q_1)\exp\left[-\mu_2 P - \frac{p_1}{\mu_3}\ln\left(\frac{g_1 + I_L^* e^{-\mu_3 P}}{g_1 + I_L^*}\right)\right]$$

and

$$\lambda_2 = (1-q_2)\exp\left[r_2(0)P - \int_0^P \frac{aE^P(t)}{g_2}dt\right]$$

with

$$E^P(t) = E^* \exp\left[-\mu_2 t - \frac{p_1}{\mu_3}\ln\left(\frac{g_1 + I_L^* e^{-\mu_3 t}}{g_1 + I_L^*}\right)\right].$$

Similarly, we have that $\lambda_1 < 1$ holds true whenever the TFPS exists (i.e. in the condition (S2.4)). Thus, we can conclude that the TFPS is locally asymptotically stable if $\lambda_2 < 1$.

Next, we consider the global stability of the TFPS, that is, the global attraction of it. Consider the following system

$$\begin{cases} \frac{dT_*}{dt} = r_2(1-bT_*)T_*, t \neq nP, \\ T_*(t^+) = (1-q_2)T_*(t), t = nP \end{cases} \tag{S2.5}$$

It is easy to verify that system (S2.5) has a periodic solution $T_*^P(t)$ with period $P$, where

$$T_*^P(t) = \frac{((1-q_2)\exp(r_2 P) - 1)\exp(r_2(t-nP))}{b\left((1-q_2)\exp(r_2 P) - 1\right)\left(\exp(r_2(t-nP)) - 1\right) + \left(\exp(r_2 P) - 1\right)}$$

for all $t \in (nP, (n+1)P]$, which is globally asymptotical stable[11]. Further, there is $dT/dt < r_2(1 - bT)T$. Thus, according to the comparison theorem of impulsive models, we have that there exists a $t^*$ and $\epsilon$ such that $T(t) < T_*^P(t) + \epsilon$ when $t > t^*$.

Denote

$$\lambda_2^* = (1 - q_2)\exp\left[r_2(0)P - \int_0^P \frac{aE^P(t)}{g_2}dt + \int_0^P \frac{aE^P(t)T_*^P(t)}{g_2(g_2 + T_*^P(t))}dt\right].$$

Assume $\lambda_2^* < 1$, then there exists an $\epsilon$ such that

$$\delta \doteq (1 - q_2)\exp\left[r_2(0)P - \int_0^P \frac{a(E^P(t) - \epsilon)}{g_2}dt + \int_0^P \frac{a(E^P(t) - \epsilon)(T_*^P(t) + \epsilon)}{g_2(g_2 + (T_*^P(t) + \epsilon))}dt\right] < 1.$$

Definitely, $\lambda_2^* < 1$ implies $\lambda_2 < 1$.

Furthermore, it is easy to see that $\frac{dI_L}{dt} > -\mu_3 I_L$. Thus, according to Theorem 1 and the comparison theorem of impulsive models, we have that there exists a $t_1^* > t^*$ such that $I_L(t) > I_L^P(t)$ when $t > t_1^*$. Similarly, there is $dE/dt \geq -\mu_2 E(t) + \frac{p_1 E(t) I_L(t)}{g_1 + I_L(t)} > -\mu_2 E(t) + \frac{p_1 E(t) I_L^P(t)}{g_1 + I_L^P(t)}$ when $t > t_1^*$. Thus, also according to Proposition 1 and the comparison theorem of impulsive models, we have that there exists a $t_2^*$ such that

$$E(t) > E^P(t) - \epsilon$$

holds when $t > t_2^* > t_1^* > t^*$. Without loss of generality, we assume that the above inequality holds for all $t \geq 0$. Hence, there is

$$\begin{cases} \frac{dT}{dt} \leq r_2(0)T - \frac{a(E^P(t)-\epsilon)T}{g_2+T}, t \neq nP, \\ T(t^+) = (1 - q_2)T(t), t = nP. \end{cases}$$

Therefore, we obtain

$$\begin{aligned} T((n+1)P) &\leq T(nP^+)\exp\left(\int_{nP}^{(n+1)P}\left(r_2(0) - \frac{a(E^P(t)-\epsilon)}{g_2+T}\right)\right) \\ &= T(nP)(1-q_2)\exp\left(r_2(0)P - \int_{nP}^{(n+1)P}\left(\frac{a(E^P(t)-\epsilon)}{g_2} - \frac{a(E^P(t)-\epsilon)T}{g_2(g_2+T)}\right)\right) \\ &< T(nP)(1-q_2)\exp\left(r_2(0)P - \int_{nP}^{(n+1)P}\left(\frac{a(E^P(t)-\epsilon)}{g_2}\right)dt \right. \\ &\quad \left. + \int_{nP}^{(n+1)P}\left(\frac{a(E^P(t)-\epsilon)(T_*^P(t)+\epsilon)}{g_2(g_2+(T_*^P(t)+\epsilon))}\right)\right) \\ &= T(nP)\delta. \end{aligned}$$

Therefore, $T((n+1)P) < T(0)\delta^{n+1}$, which means that $T((n+1)P) \to 0$ as $n \to \infty$, i.e., $T(t) \to 0$ as $t \to \infty$.

Then, we verify that $I_L(t) \to I_L^P(t)$ as $t \to \infty$. Because $T(t) \to 0$ as $t \to \infty$, thus there exist $\epsilon$ and $t^*$ such that $T(t) < \epsilon$ and $\frac{T(t)}{g_3+T(t)} < \epsilon$ when $t > t^*$. Here, we assume that $T(t) < \epsilon$ and $\frac{T(t)}{g_3+T(t)} < \epsilon$ hold true for all $t > 0$. Therefore, $\frac{dE(t)}{dt} \leq c\epsilon - \mu_2 E(t) + p_1 E(t)$. Then, by the comparison theorem of impulsive models, if $\mu_2 > p_1$, there exist an $M$ and a $t^*$ such that $E(t) < M$ when $t > t^*$. Without loss of generality, we also assume that $E(t) < M$ holds for all $t > 0$ when $\mu_2 > p_1$.

Let $\Phi_1 = |I_L(t) - I_L^P(t)|$ and taking the upper-right derivative of $\Phi_1(t)$ yields

$$\begin{aligned} D^+\Phi_1(t) &= sign(I_L(t) - I_L^P(t))(I_T'(t) - I_L^{P'}(t)) \\ &\leq -\mu_3\Phi_1(t) + \frac{p_2TE}{g_3+T} \leq -\mu_3\Phi_1(t) + p_2M\epsilon. \end{aligned}$$

It follows that

$$\begin{cases} D\Phi_1(t) \leq -\mu_3\Phi_1(t) + p_2M\epsilon, t \neq nP, \\ \Phi_1(t^+) = (1-q_3)\Phi_1(t), t = nP. \end{cases} \tag{S2.6}$$

Consequently, we have

$$\begin{aligned} \Phi_1((n+1)P) &\leq \Phi_1(nP^+)\exp\left(\int_{nP}^{(n+1)P}(-\mu_3)\right) + p_2MP\epsilon \\ &= \Phi_1(nP)(1-q_3)\exp(-\mu_3P) + p_2MP\epsilon = \Phi_1(nP)\lambda_3 + p_2MP\epsilon. \end{aligned}$$

Thus, there is $\Phi_1((n+1)P) \leq \Phi_1(0)\lambda_3^{n+1} + p_2MP\epsilon(\lambda_3^n + \lambda_3^{n-1} + ... + \lambda_3 + 1) < \Phi_1(0)\lambda_3^{n+1} + \frac{p_2MP}{(1-\lambda_3)}\epsilon$ with $\lambda_3 < 1$. This means that $\Phi_1((n+1)P) \to 0$ as $n \to \infty$ because $\epsilon$ can be small enough. That is, $I_L(t) \to I_L^P(t)$ as $t \to \infty$.

Then, we prove that $E(t) \to E^P(t)$ as $t \to \infty$. Because $I_L \to I_L^P$ and $T(t) \to 0$ when $t \to \infty$, there exists a $t^*$ such that $I_L^P - \epsilon < I_L < I_L^P + \epsilon$ and $T(t) < \epsilon$ for any positive number $\epsilon$. Without loss of generality, we assume that $I_L^P - \epsilon < I_L < I_L^P + \epsilon$ and $T(t) < \epsilon$ hold true for all $t > 0$. Let $\Phi_2 = |E(t) - E^P(t)|$ and denote $S(t) \doteq sign(E(t) - E^P(t))$. Taking the upper-right derivative of

$\Phi_2(t)$, we get

$$\begin{aligned} D^+\Phi_2(t) &= S(t)(E'(t) - {E^P}'(t)) \\ &\le -\mu_2\Phi_2(t) + cT + S(t)\left(\tfrac{p_1 I_L E}{g_1+I_L} - \tfrac{p_1 I_L^P E^P}{g_1+I_L^P}\right) \\ &\le -\mu_2\Phi_2(t) + cT + S(t)\left(\tfrac{p_1(I_L^P+S(t)\epsilon)E}{g_1+(I_L^P+S(t)\epsilon)} - \tfrac{p_1 I_L^P E^P}{g_1+I_L^P}\right) \\ &\le -\mu_2\Phi_2(t) + \tfrac{p_1(I_L^P+\epsilon)}{g_1+(I_L^P+\epsilon)}\Phi_2(t) + \tfrac{g_1 p_1 \epsilon}{(g_1+I_L^P+\epsilon)(g_1+I_L^P)} + c\epsilon. \end{aligned}$$

Further, due to $\lambda_1 < 1$, there must exist a small enough number $\epsilon$ and a big enough $t^*$ such that

$$\delta_1 \doteq (1-q_1)\exp\left(\int_{nP}^{(n+1)P}\left(-\mu_2 + \frac{p_1(I_L^P+\epsilon)}{g_1+(I_L^P+\epsilon)}\right)\right) < 1.$$

Then, by considering

$$\begin{cases} D\Phi_2(t) \le -\mu_2\Phi_2(t) + \frac{p_1(I_L^P+\epsilon)}{g_1+(I_L^P+\epsilon)}\Phi_2(t) + \frac{g_1 p_1\epsilon}{(g_1+I_L^P+\epsilon)(g_1+I_L^P)} + c\epsilon, t \ne nP \\ \Phi_2(t^+) = (1-q_1)\Phi_2(nP), t = nP. \end{cases}$$

we have

$$\begin{aligned} \Phi_2((n+1)P) &\le \Phi_2(np)(1-q_1)\exp\left(\int_{nP}^{(n+1)P}\left(-\mu_2 + \tfrac{p_1(I_L^P+\epsilon)}{g_1+(I_L^P+\epsilon)}\right)\right) \\ &+ \int_{nP}^{(n+1)P}\left(\tfrac{g_1p_1\epsilon}{(g_1+I_L^P+\epsilon)(g_1+I_L^P)} + c\epsilon\right) \le \Phi_2(nP)\delta_1 + M_1\epsilon, \end{aligned}$$

where $M_1 = \int_{nP}^{(n+1)P}\left(\frac{g_1p_1}{(g_1+I_L^P)^2} + c\right)$.

In conclusion, $\Phi_2((n+1)P) \le \Phi_2(0)\delta_1^{n+1} + M_1\epsilon(\delta_1^n + \delta_1^{n-1} + ...\delta_1 + 1) < \Phi_2(0)\delta_1^{n+1} + M_1\epsilon/(1-\delta_1)$. As $\epsilon$ is small enough when $t$ is big enough, we have $\Phi_2((n+1)P) \to 0$ as $n \to \infty$, that is, $E(t) \to E^P(t)$ as $t \to \infty$. Based on the above discussion, we have the following conclusion.

**Theorem 1.** If condition (S2.4) holds true, then system (S1.1) has a tumour free periodic solution $(E^P(t), 0, I_L^P(t))$ with period $P$. It is locally asymptotically stable when $\lambda_2 < 1$. Furthermore, if $\lambda_2^* < 1$ and $\mu_2 > p_1$ hold true, the tumour free periodic solution is globally asymptotically stable.

## 3. Baseline parameter values and parameter sets used in the main text

In order to show the stability of the periodic solution $(E^P(t), I_L^P(t))$ of subsystem (S2.2), we plot the solutions as shown in Fig.S.1 by using the baseline

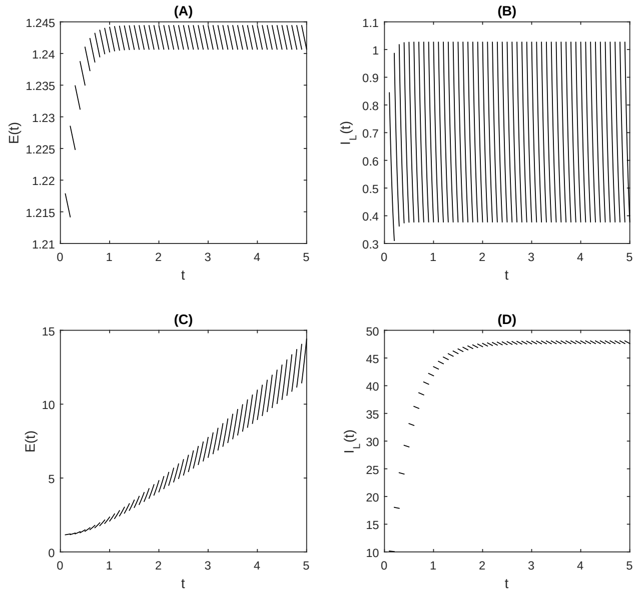


Figure S.1: Illustration of the stability of the periodic solution $(E^P(t), I_L^P(t))$ of model (S2.2). (A) The parameter values are $\mu_2 = 0.03, p_1 = 0.1245, g_1 = 2 \times 10^7, \mu_3 = 10, q_1 = 0.4, s_1 = 0.5, q_3 = 0.4, s_2 = 0.8$ with $P = 0.1$ and $\lambda_1 = 0.5928$; (B) The parameter values are $\mu_2 = 0.03, p_1 = 100, g_1 = 2 \times 10^3, \mu_3 = 0.1, q_1 = 0.2, s_1 = 0.2, q_3 = 0.2, s_2 = 10$ with $P = 0.1$ and $\lambda_1 = 1.0075$.

parameter values shown in reference (1), from which we can see that the periodic solution $(E^P(t), I_L^P(t))$ is asymptotically stable provided that the Floquet multiplier $\lambda_1 = 0.5928 < 1$, as shown in Fig.S.1(A), while it becomes unstable if $\lambda_1 = 1.0075 < 1$, as shown in Fig.S.1(B). Therefore, we focus on the parameter space at which the periodic solution $(E^P(t), I_L^P(t))$ is globally stable, otherwise the $E(t)$ will tend to infinity due to frequent administrations. The tumour-free periodic solutions and their stabilities for system (S1.1) have been shown in Fig.S.2 for various parameter settings.

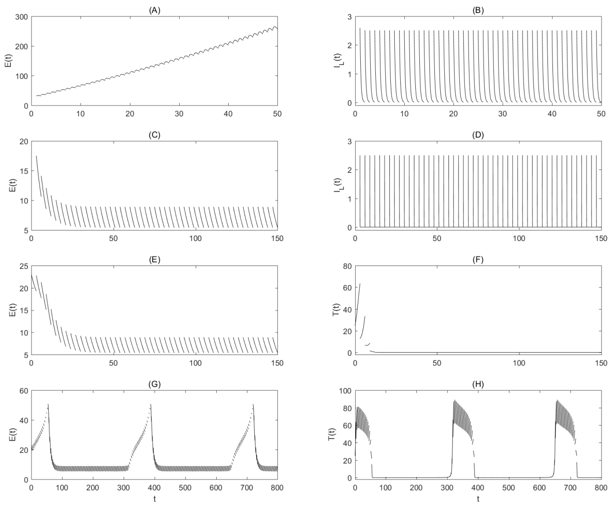

Figure S.2: The stability of the periodic solution $(E^P(t), I_L^P(t))$ of model (S2.2) and the tumour-free periodic solution $(E^P(t), 0, I_L^P(t))$ of system (S1.1). The base line parameter values are as follows: $c = 0.0128, \mu_2 = 0.1667, p_1 = 0.6917, g_1 = 70, g_2 = 5, r_2 = 1, b = 0.01, a = 0.55556, \mu_3 = 55.556, p_2 = 27.7778, g_3 = 5, q_1 = 0, s_1 = 3.5, q_2 = 0.3, q_3 = 0, s_2 = 2.5$ and $P = 3$. (A-B) The instability of $(E^P(t), I_L^P(t))$ with $g_1 = 3, g_2 = 5, \mu_3 = 5.5556, P = 1$ and $\lambda_1 = 1.0086$; (C-D) The stability of $(E^P(t), I_L^P(t))$ with $\lambda_1 = 0.6067$; (E-F) The stability of $(E^P(t), 0, I_L^P(t))$ with $q_2 = 0.8$ and $\lambda_1 = 0.3933, \lambda_2 = 0.3887$; (G-H) The instability of $(E^P(t), 0, I_L^P(t))$ with $\lambda_1 = 0.3933, \lambda_2 = 1.3605$.

The parameter set listed in Table S.1 of reference (1) is as follows:

$$0 \le c \le 0.05, \mu_2 = 0.03, p_1 = 0.1245, g_1 = 2 \times 10^7$$

$$g_2 = 1 \times 10^5, r_2 = 0.18, b = 1 \times 10^{-9}, a = 1$$

$$\mu_3 = 10, p_2 = 5, g_3 = 1 \times 10^3.$$

In order to increase the visibility of the figures shown in the main text, we make appropriate changes to get two parameter sets. For the first parameter set we let $E_0 = T_0 = I_{L0} = \frac{1}{b}$, $t_s = r_2$ and take

$$x = \frac{E}{E_0}, y = \frac{T}{T_0}, z = \frac{I_L}{I_{L0}}, \tau = t_s t, \bar{c} = \frac{cT_0}{t_s E_0}$$

$$\bar{p}_1 = \frac{p_1}{t_s}, \bar{g}_1 = \frac{g_1}{I_{L0}}, \bar{\mu}_2 = \frac{\mu_2}{t_s}, \bar{g}_2 = \frac{g_2}{T_0}, \bar{b} = bT_0$$
$$\bar{r}_2 = \frac{r_2}{t_s}, \bar{a} = \frac{aE_0}{t_s T_0}, \bar{\mu}_3 = \frac{\mu_3}{t_s}, \bar{p}_2 = \frac{p_2 E_0}{t_s I_{L0}}, \bar{g}_3 = \frac{g_3}{T_0}$$
$$\bar{s}_1 = \frac{s_1}{t_s E_0}, \bar{s}_2 = \frac{s_2}{t_s I_{L0}}.$$

The above confirms that the first parameter set used in the main text is as follows:

$$x_0 = y_0 = z_0 = 1,$$

$$c = 0.08, \mu_2 = 0.1667, p_1 = 0.6917, g_1 = 10$$

$$g_2 = 10, r_2 = 1, b = 0.03, a = 5.5556$$

$$\mu_3 = 55.556, p_2 = 27.7778, g_3 = 1.$$

Further, if we assume that $\bar{E} = N_1 E, \bar{T} = N_2 T, \bar{I}_L = N_3 I_L$, then we have

$$c \to \frac{N_1}{N_2} c, \mu_2 \to \mu_2, p_1 \to p_1, g_1 \to N_3 g_1$$

$$r_2 \to r_2, b \to \frac{1}{N_2} b, a \to \frac{N_2}{N_1} a, g_2 \to N_2 g_2$$

$$p_2 \to \frac{N_3}{N_1} p_2, g_3 \to N_2 g_3, \mu_3 \to \mu_3.$$

For scaling all three variables we choose $N_1 = 100, N_2 = 1000, N_3 = 1000$, and we have the following second parameter set employed in the main text:

$$c = 0.0128, \mu_2 = 0.1667, p_1 = 0.6917, g_1 = 70$$

$$g_2 = 5, r_2 = 1, b = 0.01, a = 0.55556$$

$$\mu_3 = 55.556, p_2 = 27.7778, g_3 = 5.$$

To fit the RCRC or IRC data sets mentioned in Section 2.4 of the main text, we got the two parameter sets listed in Table S.1.

Table S.1: The parameter sets for fitting the RCRC or IRC data sets.

| Parameter | Value ($q_2$) | Source | Value ($s_1$) | Source |
|---|---|---|---|---|
| a | 0.1005 | Estimated | 0.6417 | Estimated |
| b | 1/140 | Assumed | 1/100 | Assumed |
| c | 0.5999 | Estimated | 0.2542 | Estimated |
| r | 8.1848 | Estimated | 9.6082 | Estimated |
| $g_1$ | 22.7166 | Estimated | 70 | Assumed |
| $g_2$ | 49.3579 | Estimated | 99.9334 | Estimated |
| $g_3$ | 2.2693 | Estimated | 94.4324 | Estimated |
| $\mu_2$ | 0.6995 | Estimated | 0.4918 | Estimated |
| $\mu_3$ | 55.556 | Assumed | 13.0097 | Estimated |
| $p_1$ | 0.6917 | Assumed | 0.6917 | Assumed |
| $p_2$ | 27.7778 | Assumed | 19.3367 | Estimated |
| $q_1$ | 0 | Assumed | 0 | Assumed |
| $q_2$ | – | – | 0 | Assumed |
| $q_3$ | 0 | Assumed | 0 | Assumed |
| $s_1$ | 0 | Assumed | – | – |
| $s_2$ | 0 | Assumed | 0 | Assumed |
| $r_q$ | 0.6599 | Estimated | – | – |
| $p_s$ | – | – | 38.4268 | Estimated |

## 4. The effects of checkpoints and treatment period

A realistic scenario is that the number of tumour cells is measured at the checkpoints when the patient's cancer was diagnosed and at subsequent follow-ups, rather than being measured at the treatment point. To show this, without loss of generality we may assume that the patient has the disease checked periodically with a period $P_1$ ($P_1$ denotes the monitoring period), and the combination therapy takes place within two checkpoints $nP_1$ and $(n+1)P_1$, $n = 0, 1, 2, \cdots$. This means that there exists a positive real number $P$ satisfying $0 \leq P \leq P_1$, and at each time point $nP_1 + P$ the combination therapy is applied. Therefore,

we have the following modified model:

$$\begin{cases} \left.\begin{aligned} \frac{dE(t)}{dt} &= cT - \mu_2 E + \frac{p_1 E I_L}{g_1 + I_L}, \\ \frac{dT(t)}{dt} &= r_2(T)T - \frac{aET}{g_2 + T}, \\ \frac{dI_L(t)}{dt} &= \frac{p_2 ET}{g_3 + T} - \mu_3 I_L, \end{aligned}\right\} & t \neq nP_1 + P, \\ \left.\begin{aligned} E(t^+) &= (1 - q_1)E(t) + s_1, \\ T(t^+) &= (1 - q_2)T(t), \\ I_L(t^+) &= (1 - q_3)I_L(t) + s_2, \end{aligned}\right\} & t = nP_1 + P \end{cases} \tag{S4.7}$$

The interesting question is how the treatment point $nP_1 + P$ affects the values of monitoring points $nP_1$, i.e. how does $T(nP_1)$ (denoted by $E_n$, $T_n$ and $I_{Ln}$) change as $P$ varies from 0 to $P_1$? Moreover, we denote the values at time point $nP_1 + P$ as $E_n^P$, $T_n^P$ and $I_{Ln}^P$. Similarly, we denote $E_n^{P+}$, $T_n^{P+}$ and $I_{Ln}^{P+}$ as the values after the combination therapy. The stroboscopic maps related to time series $E_n^P$, $T_n^P$ and $I_{Ln}^P$ (or $E_n^{P+}$, $T_n^{P+}$ and $I_{Ln}^{P+}$) of model (S4.7) can be similarly defined as formula (2) in the main text, from which we can calculate the mean values of $E_n, T_n$ and $I_{Ln}$ for $n = 150, 151, \cdots, 200$ and the mean values of $E_n^P, T_n^P$ and $I_{Ln}^P$ to reveal the effects of the timing of combination therapy on RCRC and/or IRC.

First we address the effects of checkpoints and treatment period on the existence and stability of the tumour free periodic solution. To do this, let $T = 0$ and we investigate the existence of the tumour free $P_1$-periodic solution, i.e. consider the following subsystem

$$\begin{cases} \left.\begin{aligned} \frac{dE(t)}{dt} &= -\mu_2 E(t) + \frac{p_1 E(t) I_L(t)}{g_1 + I_L(t)}, \\ \frac{dI_L(t)}{dt} &= -\mu_3 I_L(t), \end{aligned}\right\} & t \neq nP_1 + P, \\ \left.\begin{aligned} E(t^+) &= (1 - q_1)E(t) + s_1, \\ I_L(t^+) &= (1 - q_3)I_L(t) + s_2, \end{aligned}\right\} & t = nP_1 + P. \end{cases} \tag{S4.8}$$

Integrating the second equation of system (S4.8) within the interval $(nP_1, nP_1+$

$P]$, we get

$$I_L(t) = \exp\left[-\mu_3(t - nP_1)\right] I_L(nP_1).$$

Hence, we have $I_L((nP_1+P)^+) = (1-q_3)I(nP_1+P)+s_2 = (1-q_3)I(nP_1)e^{-\mu_3 P}+ s_2$. Further, integrating the equation $\frac{dI_L(t)}{dt} = -\mu_3 I_L$ in the interval $(nP_1 + P, (n+1)P_1]$ with an initial condition $I((nP_1 + P)^+)$, we obtain

$$I((n+1)P_1) = I((nP_1+P)^+)e^{-\mu_3(P_1-P)} = ((1-q_3)I(nP_1)e^{-\mu_3 P}+s_2)e^{-\mu_3(P_1-P)}.$$

Let $I((n + 1)P_1) = I(nP_1)$ and solving the above equation, we can get a fixed point $I_L*$, which is given by

$$I_L^* = \frac{s_2}{e^{\mu_3(P_1-P)} - (1 - q_3)e^{-\mu_3 P}}.$$

Then, solving the equation $dI_L = -\mu_3 I_L$ with the initial condition $I_L^*$, we have $I_L^{**} = I((nP_1 + P)^+) = \frac{s_2}{1-(1-q_3)e^{-\mu_3 P_1}}$.

Thus, there exists a periodic solution of $I_L(t)$, which is given by

$$\begin{cases} I_L(t) = I_L^* e^{-\mu_3(t-nP_1)}, t \in (nP_1, nP_1 + P] \\ I_L(t) = I_L^{**} e^{-\mu_3}(t - (nP_1 + P)), t \in (nP_1 + P, (n+1)P_1). \end{cases}$$

Because there is no implementation of the control strategy at the time point $nP_1$, we can also write the periodic solution of $I_L(t)$ in the following form:

$$I_L(t) = I_L^{**} e^{-\mu_3(t-nP_1)}, t \in (nP_1 + P, (n+1)P_1 + P]. \tag{S4.9}$$

Note that, here $I_L^{**}$ is equal to the $I_L^*$ of the former model (i.e. model (S2.2)). Therefore the periodic solutions of the two models in terms of $I_L$ are the same if we take the pulse periods as the same. Further, also because there is no control at $nP_1$, we can consider the existence of the periodic solution of $E(t)$ in the interval $(nP_1+P, (n+1)P_1+P]$. As the periodic solution of $I_L$ is the same, we can obtain the same periodic solution of $E(t)$ by substituting the periodic solution of $I_L$ into model (S2.2). All these results confirm that if we fix the treatment period, then the treatment time point does not affect the existence and stability of the tumour free periodic solution. Thus, the interesting question is how does the number of tumour cells measured at checkpoints during the monitoring of a

patient after diagnosis rather than at the treatment time influence the dynamics of tumour cells? To address this, we carry out some numerical investigations about this in the following.

If we fix the parameter values as those shown in Fig.S.3 and let $P$ increase from 3 to 17, then the time series of $E(t)$ and $T(t)$ have been generated in each subplot. Although the whole solution curves seem to be similar, the phase positions of the extreme points of each solution curve change greatly as the time points $P$ of combined treatment vary. To reveal the effects in more detail, we plot various outputs in Fig.S.4 for the different combined treatment points, i.e. $P = 5$ or 11. The time series have been shown in Fig.S.4(A) and (B); The number of effector cells and tumour cells at the each checkpoint $nP_1$ are shown in Fig.S.4(C), and the number of effector cells and tumour cells at the each monitoring point $nP_1 + P$ are shown in Fig.S.4(D). While the relations between $E_n$ and $E_{n+1}$, $T_n$ and $T_{n+1}$, $E_n^P$ and $E_{n+1}^P$, $T_n^P$ and $T_{n+1}^P$ are shown in Fig.S.4(E) and (F) for $P = 5$, from which we can see that the differences are obvious including the maximum amplitudes and iteration patterns. Similar outputs are shown in Fig.S.4(G-J) for $P = 11$.

The difference in the number of tumour cells between the monitoring point and the treatment point can also be discussed through bifurcation analyses, as shown in Fig.S.5 for one parameter bifurcation diagrams with respect to $q_2$, and in Fig.S.6 for two-parameter bifurcation diagrams with respect to $q_2$ and $P$. One parameter bifurcation diagrams reveal that the numbers of tumour cells in the monitoring site and the treatment site are very different, and the bi-stable regions also change slightly, as shown in Fig.S.5(K) and (L), which indicate that the RCRCs can be significantly affected if we record the number of tumour cells at different time points. But we can see that the values of $T_n$ (i.e. measures of the number of tumour cells before the treatment) do not change too much as the treatment period, $P$, changes.

However, the two-parameter bifurcation diagrams shown in Fig.S.6 reveal that the numbers of tumour cells detected at two different time points may change significantly with the change of parameters. There is no doubt that the

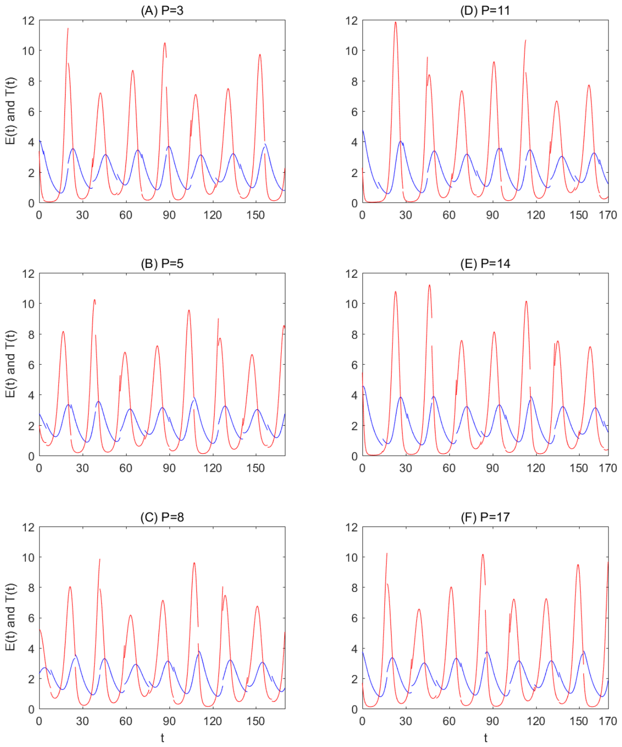


Figure S.3: Illustration of the effect of checkpoint and treatment period on the solutions of model (S4.7). The baseline parameter values are as follows: $c = 0.08, \mu_2 = 0.1667, p_1 = 0.6917, g_1 = 10, g_2 = 10, r_2 = 1, b = 0.03, a = 5.5556, \mu_3 = 55.556, p_2 = 27.7778, g_3 = 1, q_1 = 0.08, s_1 = 0.5, q_2 = 0.2, q_3 = 0.08, s_2 = 0.5$, $P_1 = 17$ with $P$ varying as shown in each subplot. Blue and red indicate the solutions of $E(t)$ and $T(t)$ in each subplot, respectively.

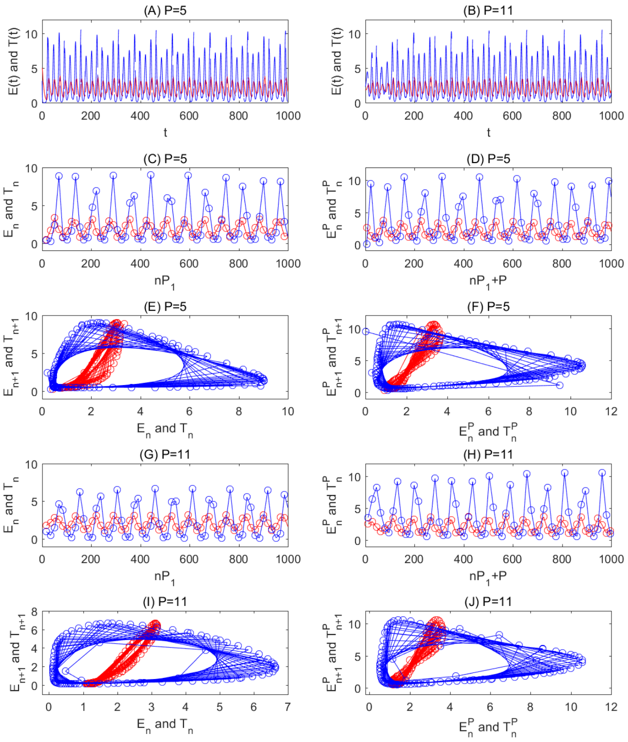


Figure S.4: Illustration of the effect of checkpoint and treatment period on the solutions of model (S4.7). The baseline parameter values are as follows: $c = 0.08, \mu_2 = 0.1667, p_1 = 0.6917, g_1 = 10, g_2 = 10, r_2 = 1, b = 0.03, a = 5.5556, \mu_3 = 55.556, p_2 = 27.7778, g_3 = 1, q_1 = 0.08, s_1 = 0.5, q_2 = 0.2, q_3 = 0.08, s_2 = 0.5,$ $P_1 = 17$ with $P = 5$ or $P = 11$ as shown in each subplot. Red and blue indicate the solutions of $E(t)$ and $T(t)$ in each subplot, respectively.

huge difference in monitoring or detecting tumour cells at different times will have a great impact on the treatment plan, and then on the treatment effect. Therefore, it is very important to design a reasonable treatment and detection scheme for the treatment of tumours.

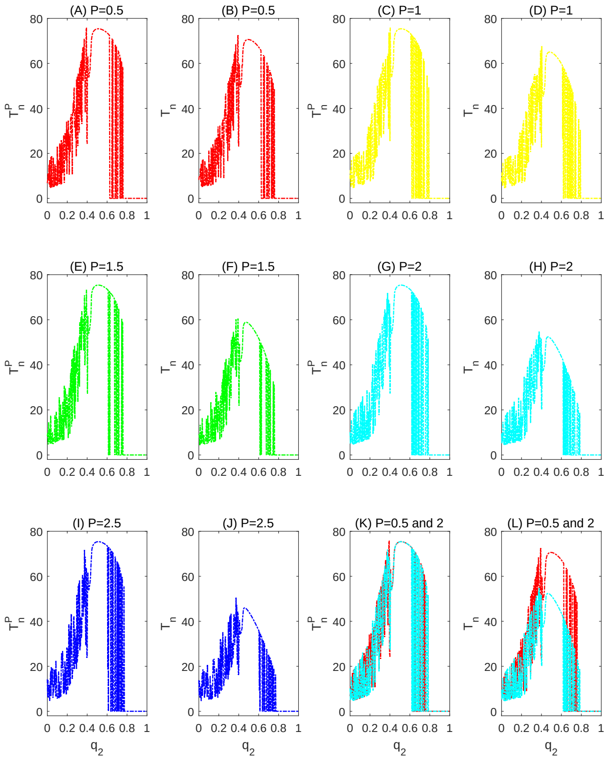


Figure S.5: Bifurcation diagrams with respect to killing rate $q_2$ to reveal the effect of checkpoint and treatment periods on the RCRCs. The baseline parameter values are as follows: $c = 0.0128, \mu_2 = 0.1667, p_1 = 0.6917, g_1 = 70, g_2 = 5, r_2 = 1, b = 0.01, a = 0.55556, \mu_3 = 55.556, p_2 = 27.7778, g_3 = 5, q_1 = 0, s_1 = 3, q_3 = 0, s_2 = 3,$ $P_1 = 3$ with $P$ varying as shown in each subplot.

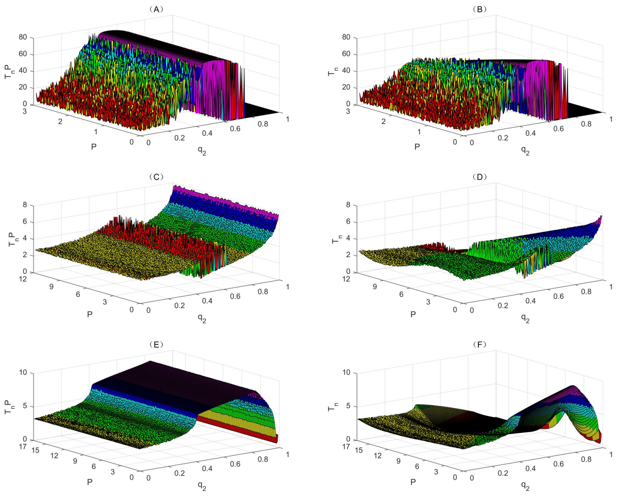


Figure S.6: Bifurcation diagrams with respect to killing rate $q_2$ and treatment point $P$ to reveal the effect of checkpoint and treatment periods on the tumour development. The baseline parameter values for (A) and (B) are as follows: $c = 0.0128, \mu_2 = 0.1667, p_1 = 0.6917, g_1 = 70, g_2 = 5, r_2 = 1, b = 0.01, a = 0.55556, \mu_3 = 55.556, p_2 = 27.7778, g_3 = 5, q_1 = 0, s_1 = 3, q_3 = 0, s_2 = 3$, $P_1 = 3$ with $P$ varying from 0 to 3. The baseline parameter values for (C-F) are as follows: $c = 0.08, \mu_2 = 0.1667, p_1 = 0.6917, g_1 = 10, g_2 = 10, r_2 = 1, b = 0.03, a = 5.5556, \mu_3 = 55.556, p_2 = 27.7778, g_3 = 1, q_1 = 0.08, s_1 = 0.5, q_2 = 0.2, q_3 = 0.08, s_2 = 0.5$, $P_1 = 12$ in (C) and (D), $P_1 = 17$ in (E) and (F).

**References**


1. Kirschner D, Panetta JC. Modeling immunotherapy of the tumor-immune interaction. J. Math. Biol. 1998; 37:235-252.
2. Lakmechea A, Arino O. Nonlinear mathematical model of pulsed-therapy of heterogeneous tumors. Nonlinr. Anal. RWA. 2001; 2:455-465 (2001).
3. Bunimovich-Mendrazitsky S, Byrne H, Stone L. Mathematical model of pulsed immunotherapy for superficial bladder cancer. Bull. Math. Biol. 2008; 70: 2055-2076.
4. Cappuccio A, Elishmereni M, Agur Z. Cancer immunotherapy by interleukin-21: potential treatment strategies evaluated in a mathematical model. Cancer Res. 2006; 66:7293C00.
5. de Pillis LG, Radunskaya AE, Wiseman CL. A validated mathematical model of cell-mediated immune response to tumor growth. Cancer Res. 2005; 65:7950C58.
6. Serre R, Benzekry S, Padovani L, Christophe Meille C, Andre N, Ciccolini J, et al. Mathematical modeling of cancer immunotherapy and its synergy with radiotherapy. Cancer Res. 2016; 76:4931C40.
7. Wei H, Lin J. Periodically pulsed immunotherapy in a mathematical model of tumor-immune interaction. Int. J. Bif. Chaos 2013; 23:1350068.
8. Yang J, Tang SY, Cheke RA. Modelling pulsed immunotherapy of tumour-immune interaction. Math. Comp. Sim. 2015; 109:92-112.
9. Kovacs E. The serum levels of IL-12 and IL-16 in cancer patients relation to the tumour stage and previous therapy. Biomed. Pharmacother. 2001; 55:111-116.
10. Xian JM, Yang H, Lin YH, Liu SX. Combination nonviral murine interleukin 2 and interleukin 12 gene therapy and radiotherapy for head and neck squamous cell carcinoma. Arch. Otolaryngol. Head Neck. Surg. 2005; 131:1079-1085.
11. Liang JH, Tang SY. Optimal dosage and economic threshold of multiple pesticide applications for pest control. Math. Comp. Mod. 2010; 51: 487-503.
